# MicroRNA-889 Inhibits Autophagy To Maintain Mycobacterial Survival in Patients with Latent Tuberculosis Infection by Targeting TWEAK

**DOI:** 10.1128/mBio.03045-19

**Published:** 2020-01-28

**Authors:** Der-Yuan Chen, Yi-Ming Chen, Chin-Fu Lin, Che-Min Lo, Hung-Jen Liu, Tsai-Ling Liao

**Affiliations:** a Department of Internal Medicine, Taichung Veterans General Hospital, Taichung, Taiwan; b Rheumatology and Immunology Center, China Medical University Hospital, Taichung, Taiwan; c Translational Medicine Laboratory, Rheumatology and Immunology Center, China Medical University Hospital, Taichung, Taiwan; d College of Medicine, China Medical University, Taichung, Taiwan; e Department of Medical Research, Taichung Veterans General Hospital, Taichung, Taiwan; f Rong Hsing Research Center for Translational Medicine, National Chung Hsing University, Taichung, Taiwan; g Ph.D. Program in Translational Medicine, National Chung Hsing University, Taichung, Taiwan; h Department of Pathology and Laboratory Medicine, Taichung Veterans General Hospital, Taichung, Taiwan; i Institute of Molecular Biology, National Chung Hsing University, Taichung, Taiwan; j The iEGG and Animal Biotechnology Center, National Chung Hsing University, Taichung, Taiwan; Sequella, Inc.

**Keywords:** autophagy, biomarker, latent tuberculosis infection (LTBI), microRNA-889, tumor necrosis factor-like weak inducer of apoptosis (TWEAK)

## Abstract

Autophagy plays an important role in protecting the host against pathogens. Mycobacterium tuberculosis can suppress autophagy and then remain dormant and survive within the host for an extended period, which is responsible for latent tuberculosis infection (LTBI). Here, we explored the role of microRNAs (miRNAs) in LTBI. The miRNA profiles were explored using the next-generation sequencing approach, followed by quantitative reverse transcription-PCR validation. The biological function of candidate miRNA was evaluated using immunoblotting, immunofluorescence techniques, and enzyme-linked immunosorbent assay in an *in vitro* human TB granuloma model. An increased miR-889 expression was observed in patients with LTBI compared with that in patients without infection. The reporter assay identified tumor necrosis factor (TNF)-like weak inducer of apoptosis (TWEAK) as the target of miR-889. Mycobacterial infection induced TWEAK upregulation in the early phase. TWEAK induced autophagy and promoted mycobacterial autophagosome maturation through activation of AMP-activated protein kinase (AMPK). Upon entry to LTBI status, elevated miR-889 levels were associated with TNF alpha (TNF-α) and granuloma formation/maintenance. MiR-889 inhibited autophagy via posttranscriptional suppression of TWEAK expression to maintain mycobacterial survival in granulomas. Adalimumab (anti-TNF-α monoclonal antibody) treatment reduced levels of both TNF-α and miR-889 and caused granuloma destruction and LTBI reactivation. The circulating miR-889 and TWEAK levels were correlated with LTBI and subsequently associated with anti-TNF-α-related LTBI reactivation in patients. We propose that miR-889 and TWEAK can act as targets that can be manipulated for antimycobacterial therapeutic purposes and act as candidate biomarkers for LTBI and LTBI reactivation, respectively.

## Abstract (precis)

TB remains a leading cause of morbidity and mortality worldwide. Approximately one-quarter of the world’s population has latent TB infection. TWEAK is a multiple-function cytokine and may be used as a target for the treatment of rheumatic diseases, cardiovascular diseases, and renal diseases. Here, we demonstrated a novel relationship between TWEAK and activation of the autophagic machinery which promotes antimycobacterial immunity. Additionally, TB infection is highly dynamic and determined by the interaction between the host and mycobacterium. We demonstrated a mechanism of fine-tuned balance between the mycobacterium and host for granuloma formation and/or maintenance in LTBI status. Once patients entered LTBI status, the upregulation of miR-889 was associated with TNF-α levels and granuloma formation to maintain mycobacterial survival. Adalimumab (a TNF-α inhibitor) reduced both TNF-α and miR-889 levels and caused LTBI reactivation and, thus, TWEAK enhancement. MiR-889 and TWEAK may become potential diagnostic biomarkers or therapeutic targets for LTBI and LTBI reactivation, respectively.

## INTRODUCTION

Autophagy is a natural defense mechanism that is conducted to eliminate microbial infection, playing a crucial role in controlling the fate of invading pathogens (1). Tuberculosis (TB) is a major cause of morbidity and mortality worldwide. Mycobacterium tuberculosis has developed a mechanism that prevents the autophagy of immune cells so that it can survive in host cells and remain dormant for a longer period, which is responsible for latent TB infection (LTBI) (2). Most individuals infected with M. tuberculosis have an LTBI, and this population is an important reservoir for disease reactivation (3). Increased evidence indicates an elevated TB risk in patients with rheumatoid arthritis (RA) (4, 5); the risk is even higher for those receiving anti-tumor necrosis factor alpha (TNF-α) therapy (6). Gardam et al. (7) revealed that active TB in RA patients receiving anti-TNF-α therapy appears to be largely caused by LTBI reactivation. The tuberculin skin test (TST) and interferon gamma (IFN-γ) release assays (IGRAs) are currently the commonly used methods to screen for LTBI (8). However, the clinical utility of TST is not reliable in bacillus Calmette-Guérin (BCG)-vaccinated patients (9), and it has a low specificity. Although the specificity of IGRAs is enhanced, the cost of IGRAs is high. Additionally, neither the TST nor IGRAs can discriminate between LTBI and TB disease (10).

MicroRNAs (miRNAs) are key regulators that posttranscriptionally repress the expression of target mRNAs (11). miRNAs can be detected in body fluids and are emerging as novel disease biomarkers (12). Accumulating validations demonstrate that miRNAs are regulators during mycobacterial infections and can be potential biomarkers for the diagnosis of TB diseases (13, 14). Most of these miRNAs were involved in TB pathogenesis by targeting autophagy-related genes (ATGs) or regulating autophagic activity, and thus mycobacterial survival was maintained. However, the role of miRNAs in modulating host defenses in the LTBI period and their clinical relevance are unclear.

In the present study, we investigated the candidate miRNAs that were significantly associated with LTBI. We also explored the biological roles of the candidate miRNAs using a cell-based assay and an *in vitro* human TB granuloma model. Finally, we investigated the pathogenic role of candidate miRNAs in LTBI and LTBI reactivation.

## RESULTS

### Clinical characteristics of RA patients.

A total of 120 participants, including 97 RA patients and 23 healthy subjects, were enrolled. Among the RA patients, 35 with LTBI had positive QuantiFERON-TB Gold (QFT-G) results, 12 had nontuberculous mycobacterial (NTM) diseases, and 50 did not have mycobacterial infections. There were no significant differences in ages, proportions of females, rates of positivity for rheumatoid factor or anti-citrullinated peptide antibodies, DAS28 values, daily dosages of corticosteroids, proportions of disease-modifying antirheumatic drugs used, or frequencies of comorbidity among RA patients with LTBI, NTM, and those without infection (see Table S1 in the supplemental material).

10.1128/mBio.03045-19.2 TABLE S1 Demographic data and laboratory findings for rheumatoid arthritis (RA) patients with latent tuberculosis infection (LTBI), with nontuberculous-mycobacterium (NTM) infection, and without infection. Download Table S1, DOCX file, 0.01 MB. Copyright © 2020 Chen et al. 2020 Chen et al. This content is distributed under the terms of the Creative Commons Attribution 4.0 International license.

### Differentially expressed miRNAs using NGS and quantitative reverse transcription-PCR (QRT-PCR).

**FIG 1:**
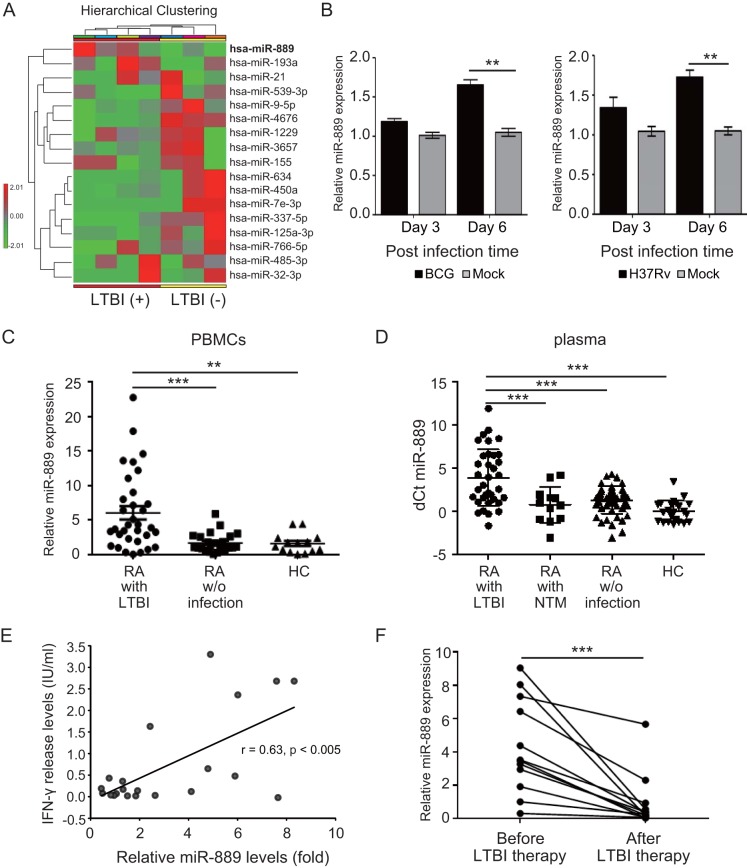
Increased microRNA-889 (miR-889) levels in patients with latent tuberculosis infection (LTBI). (A) Differentially expressed miRNAs in peripheral blood mononuclear cells (PBMCs) from rheumatoid arthritis (RA) patients with and without LTBI were identified using next-generation sequencing (NGS) analysis. A hierarchical clustering of miRNA profiles in patients with and without the LTBI group is shown. The relative expression levels of miRNAs are depicted according to a color scale (red represents a relative expression greater than the median expression level across all samples, and green represents an expression level lower than the median). (B) Human PBMCs were infected with M. bovis BCG (left) or M. tuberculosis H37Rv (right) at an MOI of 0.1 for the indicated times, and the expression levels of miR-889 were measured by quantitative reverse transcription-PCR (QRT-PCR). (C) Validation of miR-889 expression in PBMCs from RA patients with or without LTBI and healthy controls using QRT-PCR. (D) Increased circulating miR-889 levels in plasma from RA patients with LTBI determined by a QRT-PCR assay. The Δ*C_T_* was calculated as follows: the mean for healthy controls minus the value for a patient (*C_T_*_miRNA gene_ – *C_T_*_Rnu6/cel-miR-39_). (E) The expression of miR-889 was positively correlated with IFN-γ release levels in the QFT-G assay of LTBI patients. (F) Decreases in miR-889 expression levels paralleled the clinical remission in LTBI patients after 3 months of therapy. All experiments were performed in triplicate, and data are presented as means ± SEMs. **, *P < *0.01; ***, *P < *0.005.

In the first stage of miRNA next-generation sequencing (NGS) analysis, 4 out of 7 RA patients had LTBIs and 3 were noninfected subjects. After normalization, we observed 17 miRNAs that were distinctively expressed in peripheral blood mononuclear cells (PBMCs); 6 miRNAs were upregulated and 11 miRNAs were downregulated in LTBI patients compared with their levels of expression in noninfected patients (Fig. 1A; Table S2). Among the six upregulated miRNAs, miR-889 exhibited the highest fold change, 4.02-fold. miR-889 expression increased in human PBMCs with M. bovis BCG infection (*P < *0.01) (Fig. 1B, left) or M. tuberculosis H37Rv (*P < *0.01) (Fig. 1B, right) at a multiplicity of infection (MOI) of 0.1, and the expression level was time dependent. Additionally, miR-889 expression increased after mycobacterial infection in a dose-dependent manner (Fig. S1A). Therefore, we focused on miR-889 for the further validation.

10.1128/mBio.03045-19.3 TABLE S2 Differentially expressed miRNAs in PBMCs from RA patients with LTBI compared with those without infection, identified by miRNA NGS analysis. Download Table S2, DOCX file, 0.01 MB. Copyright © 2020 Chen et al. 2020 Chen et al. This content is distributed under the terms of the Creative Commons Attribution 4.0 International license.

10.1128/mBio.03045-19.4 FIG S1 Human PBMCs were infected with M. bovis BCG or M. tuberculosis H37Rv at the indicated MOIs for 24 h. (A and B) The intracellular miR-889 (A) and secretory TWEAK (B) levels were measured using QRT-PCR and ELISA, respectively. (C) The level of secretory TWEAK was negatively correlated with IFN-γ release levels in the QFT-G assay of LTBI patients. All experiments were performed in triplicate, and data are presented as means ± SEMs. *, *P < *0.05; **, *P < *0.01. Download FIG S1, TIF file, 2.2 MB. Copyright © 2020 Chen et al. 2020 Chen et al. This content is distributed under the terms of the Creative Commons Attribution 4.0 International license.

### Association of circulating miR-889 levels with LTBI.

There was a significantly higher expression of miR-889 in the available PBMCs from RA patients with LTBI (*n* = 33; mean ± standard error of the mean [SEM], 6.06-fold ± 0.97-fold) (Fig. 1C) than in those without infection (*n* = 24; 1.71-fold ± 0.28-fold; *P < *0.005) and healthy controls (*n* = 14; 1.62 ± 0.38-fold; *P < *0.01).

To verify whether miR-889 was a potential biomarker for LTBI in RA patients, we examined plasma miR-889 levels. The QRT-PCR results (Fig. 1D) showed significantly higher miR-889 levels in patients with LTBI (*n* = 35; change in threshold cycle [Δ*C_T_*], 3.90 ± 0.56) than in those with NTM infection (*n* = 12; Δ*C_T_*, 0.77 ± 0.60; *P < *0.005), patients with RA without infection (*n* = 50; Δ*C_T_*, 1.31 ± 0.23; *P < *0.005), and healthy controls (*n* = 23; Δ*C_T_*, 0.00 ± 0.26; *P < *0.005). Moreover, we demonstrated a positive correlation between miR-889 expression levels and released IFN-γ levels in a QFT-G assay of LTBI patients (*r* = 0.63, *P < *0.005) (Fig. 1E). A decrease of miR-889 expression (*n* = 12, 4.31-fold ± 0.81-fold versus 0.92-fold ± 0.47-fold, *P < *0.005) was observed in LTBI patients after prophylactic therapy (Fig. 1F).

### MiR-889 targets TWEAK.

**FIG 2:**
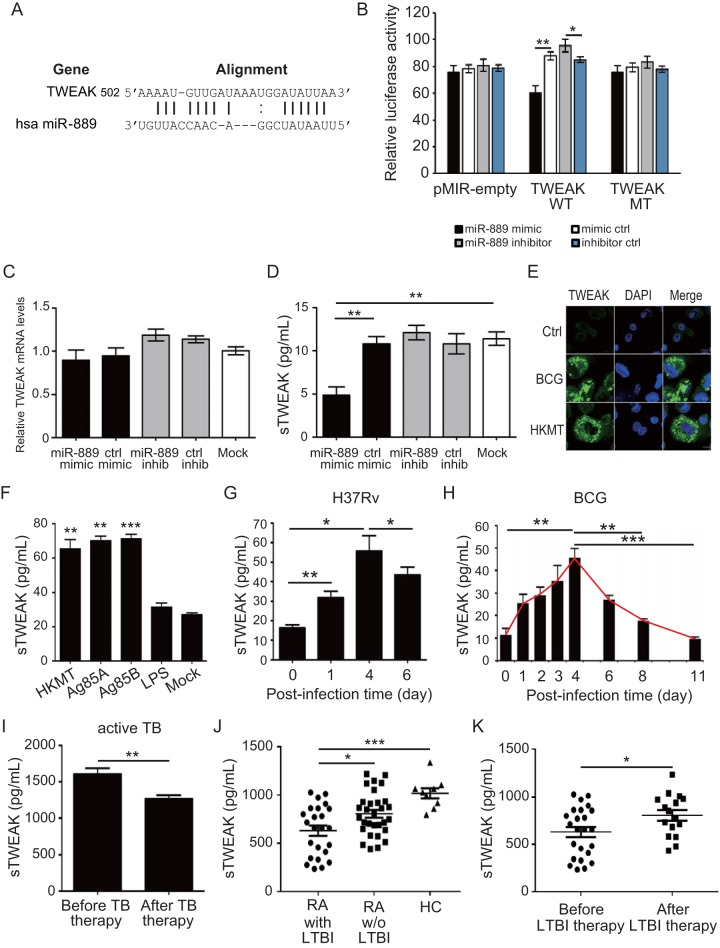
miR-889 targets tumor necrosis factor-like weak inducer of apoptosis (TWEAK). (A) The potential target for miR-889 was TWEAK. Sequence alignment of miR-889 with reverse complementary TWEAK (http://www.microrna.org). hsa, *Homo sapiens*. (B) 293T cells were cotransfected with a wild-type (WT) TWEAK 3′UTR, a mutant (MT) TWEAK 3′UTR, or an empty pMIR-REPORT vector and pTK-RL plasmid, together with a miR-889 mimic/mimic control or a miR-889 inhibitor/inhibitor control. After 36 h, luciferase activities were measured and normalized by determining Renilla luciferase activity. (C and D) Comparison of the levels of mRNA (C) or soluble TWEAK (D) released from THP-1 cells transfected with the miR-889 mimic, miR-889 inhibitor, mimic/inhibitor control, or nontransfected cells (mock). ctrl, control; inhib, inhibitor. (E to K) *Mycobacterium* induced TWEAK upregulation in early infection, and then TWEAK expression declined in patients with LTBI. (E) Dramatically enhanced intracellular TWEAK levels in THP-1 cell-derived macrophages after M. bovis BCG infection (MOI = 10) or heat-killed M. tuberculosis (HKMT) treatment (10 μg/ml) for 24 h using an immunofluorescence assay. (F) Analysis of secretory TWEAK (sTWEAK) levels in human PBMCs in response to the stimulation of HKMT treatment, M. tuberculosis secretory proteins (Ag85A or Ag85B, 4 μg/ml), and lipopolysaccharides (LPS) from Escherichia coli 055:B5 treatment by using an ELISA. (G and H) The kinetics of secretory TWEAK levels from human PBMCs with M. tuberculosis H37Rv (G) or M. bovis BCG (H) infection (MOI = 0.1) was measured by using an ELISA. (I) The dynamic change of TWEAK levels in serum was positively correlated with M. tuberculosis infection that paralleled the clinical remission in patients after anti-TB therapy. (J) Comparison of expression levels of secretory TWEAK in RA patients with and without (w/o) LTBI and healthy controls (HC). (K) Increases in secretory TWEAK levels in LTBI patients after anti-LTBI therapy. All experiments were performed in triplicate, and data are presented as means ± SEMs. *, *P < *0.05; **, *P < *0.01; ***, *P < *0.005.

Bioinformatics analysis (http://www.microrna.org) (15) revealed TNF-like weak inducer of apoptosis (TWEAK; GenBank accession number NM_003809) as a potential seed match for miR-889 in its 3′ untranslated region (3′UTR) (Fig. 2A). To validate whether TWEAK is the target of miR-889, a luciferase reporter plasmid was constructed by cloning the predicted seed sequence in the human TWEAK 3′UTR into the pMIR-REPORT luciferase vector; the plasmid with the mutation at the putative binding site was used as a control. Our results showed that miR-889 mimics significantly decreased (*P < *0.01), while those of miR-889 inhibitors significantly enhanced, the luciferase activity in cells transfected with the TWEAK 3′UTR plasmid, compared to that of the cells transfected with the inhibitor control (*P < *0.05). No significant change in luciferase activity was observed in cells transfected with the mutant TWEAK 3′UTR construct or the pMIR-REPORT plasmid (Fig. 2B), indicating that TWEAK is the target of miR-889 and may be negatively regulated by miR-889. No significant difference in levels of TWEAK mRNA expression existed between miR-889 mimic-expressing cells and control cells or nontransfected cells (Fig. 2C). However, a decreased expression of TWEAK protein levels (Fig. 2D) was detected in miR-889-overexpressing cells compared with that in control cells (*P < *0.01), indicating that TWEAK may be negatively posttranscriptional regulated by miR-889.

### *Mycobacterium*-induced TWEAK was upregulated in early infection and then declined in patients with LTBI.

Next, we validated the association between TWEAK expression and mycobacterial infection. TWEAK expression increased after mycobacterial infection in a dose-dependent manner (Fig. S1B). Increased intracellular (Fig. 2E) and secretory (Fig. 2F) TWEAK levels are illustrated in THP-1 cells and human PBMCs treated with heat-killed M. tuberculosis (HKMT; 10 μg/ml) or M. tuberculosis secretory proteins (Ag85A and Ag85B; 4 μg/ml), suggesting that the mycobacterial component might contribute to TWEAK upregulation. We analyzed the dynamics of TWEAK expression during LTBI by using the *in vitro* human TB granuloma model. An increased TWEAK level was detected early at day 4 of infection (H37Rv patients, 55.7 ± 7.8 versus 16.3 ± 1.5 pg/ml, *P < *0.05 [Fig. 2G]; BCG patients, 45.7 ± 4.1 versus 11.3 ± 3.0 pg/ml, *P < *0.01 [Fig. 2H]) and then declined after that day, and a granuloma-like structure developed at day 8. The results revealed that TWEAK was induced at early infection and then declined in patients with the LTBI status.

To support the findings with a cell-based assay, we examined the TWEAK levels in the serum of one patient with M. tuberculosis infection before and after anti-TB therapy. The results showed higher levels of TWEAK (1,607.7 ± 79.2 pg/ml) (Fig. 2I) in this patient at the time of active TB status (based on a positive TB culture result) than in healthy controls (1,017.0 ± 52.5 pg/ml) (Fig. 2J). After anti-TB therapy, a significantly decreased TWEAK level was measured, compared to that before therapy (1,263.8 ± 49.8 pg/ml, *P < *0.01) (Fig. 2I). We further validated the association of TWEAK levels with LTBI in RA patients and revealed significantly lower levels of TWEAK in LTBI patients (630.1 ± 53.2 pg/ml) than in noninfected patients (805.1 ± 40.7 pg/ml, *P < *0.05) and healthy controls (1,017.0 ± 52.5 pg/ml, *P < *0.005) (Fig. 2J). Additionally, we demonstrated a negative correlation between TWEAK levels and released IFN-γ levels in the QFT-G assay of LTBI patients (γ = 0.43, *P < *0.005) (Fig. S1C). Similarly, TWEAK levels were elevated in LTBI patients after anti-LTBI therapy (630.1 ± 53.2 pg/ml versus 805.7 ± 55.6 pg/ml, *P < *0.05) (Fig. 2K).

### TWEAK induces autophagy and autophagosome formation in macrophages.

**FIG 3:**
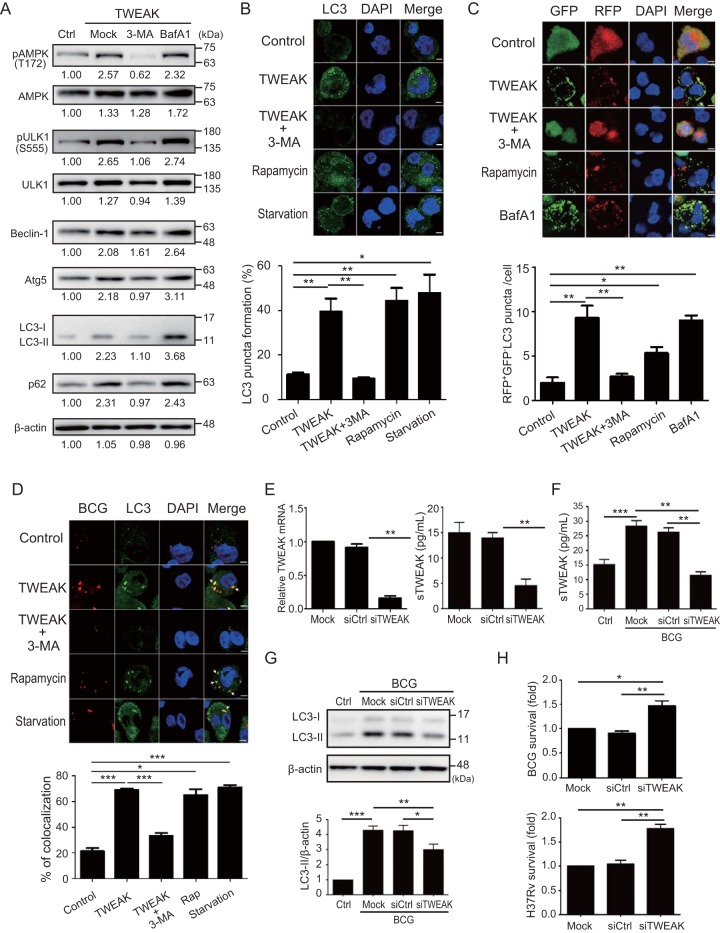
TWEAK induces autophagy through AMPK activation and promotes mycobacterial autophagosome maturation to inhibit mycobacterial survival. (A) THP-1 cell-derived macrophages were treated with TWEAK (100 ng/ml) in the absence or presence of 3-MA (10 μM) or bafilomycin A1 (100 nM) for 24 h. The phosphorylated AMPK at Thr172 (pAMPK), total AMPK, phosphorylated ULK1 at Ser555 (pULK1), total ULK1, Beclin-1, Atg5, LC3, p62, or β-actin levels were detected by an immunoblotting analysis. (B) THP-1 cell-derived macrophages were treated with the indicated reagent for 24 h. Endogenous LC3 was stained with LC3 antibody (green), and LC3 puncta were detected by confocal microscopy and quantified. (C) THP-1 cells stably expressing RFP-GFP-LC3 fusion protein were treated with the indicated reagent for 24 h, and RFP-GFP-LC3 puncta were detected by confocal microscopy and quantified. (D) THP-1 cell-derived macrophages were treated with the indicated reagent for 24 h and then infected with Texas Red-labeled M. bovis BCG for 2 h and stained with LC3 antibody. LC3 puncta (green) and BCG (red) were detected by confocal microscopy (upper panel). The fraction (percentage) of mycobacterium-containing phagosomes colocalizing with LC3 puncta was determined (lower panel). (E) THP-1 cell-derived macrophages were transfected with control siRNA (siCtrl) or TWEAK siRNA (siTWEAK) (30 nM) for 24 h. To assess the knockdown efficiency, mRNA (left) and protein (right) levels of TWEAK expression were detected by QRT-PCR and an ELISA, respectively. (F to H) TWEAK knockdown cells were infected with M. bovis BCG or M. tuberculosis H37Rv at an MOI of 10 for 24 h. The levels of secretory TWEAK (F) and LC3 or β-actin (G) were detected by an ELISA and immunoblotting, respectively. The ratios of LC3-II to β-actin were calculated. (H) Mycobacterial survival in TWEAK knockdown cells was determined by a CFU assay. (Upper panel) M. bovis BCG; (lower panel) M. tuberculosis H37Rv. All experiments were performed in triplicate, and data are presented as means ± SEMs. The scale bar in the IFA image represents 5 μm. *, *P < *0.05; **, *P < *0.01; ***, *P < *0.005.

Autophagy is a major defense mechanism inhibiting mycobacterial survival in infected macrophages. Bhatnagar et al. (16) demonstrated that TWEAK augments the expression of ATGs in myotubes. To verify whether TWEAK induces autophagy, THP-1 cell-derived macrophages were treated with TWEAK (100 ng/ml) and the levels of autophagy-related LC3 were measured by immunoblotting. We revealed that TWEAK could induce autophagy in macrophages in dose- and time-dependent manners (Fig. S2). The autophagy inhibitor 3-methyladenine (3-MA; 5 mM) rescued TWEAK-induced autophagy, while bafilomycin A1 (luminal acidification and autophagosome degradation inhibitor, 100 nM) increased LC3-II expression in cells treated with TWEAK (Fig. 3A and Fig. S3C). We further dissected the association of TWEAK with the expression of other ATGs and revealed that TWEAK induced autophagy through an activation of AMP-activated protein kinase (AMPK) at Thr172 (Fig. 3A and Fig. S3A). The effect of TWEAK on AMPK activation was validated by using AMPK inhibitor (dorsomorphin, 20 μM) treatment. As shown in Fig. S4, dorsomorphin treatment significantly suppressed both phosphorylated-TWEAK-induced levels of AMPK (0.70-fold ± 0.11-fold versus 2.54-fold ± 0.29-fold, *P < *0.01) (Fig. S4B) and LC3-II expression (1.01-fold ± 0.16-fold versus 2.67-fold ± 0.18-fold, *P < *0.01) (Fig. S4C).

10.1128/mBio.03045-19.5 FIG S2 TWEAK induces autophagy in macrophages. THP-1 cell-derived macrophages were treated with the indicated concentrations of TWEAK for 24 h (A) and TWEAK (100 ng/ml) for the indicated time (B). The LC3, Atg5, Beclin-1, phosphorylated ULK1 (Ser555 and Ser757), ULK1, phosphorylated AMPK(Thr172), AMPK, and β-actin levels were detected by using immunoblotting analysis, and the ratios of LC3-II to β-actin, pULK1(S555) to ULK1, and pAMPK(T172) to AMPK were calculated as shown in the representative blots. All experiments were performed in triplicate, and data are presented as means ± SEMs. *, *P < *0.05; **, *P < *0.01. Download FIG S2, TIF file, 2.8 MB. Copyright © 2020 Chen et al. 2020 Chen et al. This content is distributed under the terms of the Creative Commons Attribution 4.0 International license.

10.1128/mBio.03045-19.6 FIG S3 THP-1 cell-derived macrophages were treated with TWEAK (100 ng/ml) in the absence or presence of 3-MA (10 μM) or bafilomycin A1 (100 nM) for 24 h. The phosphorylated AMPK at Thr172 (pAMPK), total AMPK, phosphorylated ULK1 at Ser555 (pULK1), total ULK1, LC3, and β-actin levels were detected by an immunoblotting analysis. The ratios of pAMPK to total AMPK (A), pULK1 to total ULK1 (B), and LC3-II to β-actin (C) were calculated as shown in the representative blots. All experiments were performed in triplicate, and data are presented as means ± SEMs. *, *P < *0.05; **, *P < *0.01. Download FIG S3, TIF file, 0.6 MB. Copyright © 2020 Chen et al. 2020 Chen et al. This content is distributed under the terms of the Creative Commons Attribution 4.0 International license.

10.1128/mBio.03045-19.7 FIG S4 TWEAK induces autophagy through AMPK activation. (A) THP-1 cell-derived macrophages were treated with TWEAK (10 or 100 ng/ml) alone or TWEAK combined with dorsomorphin (20 μM) for 24 h. The pAMPK, total AMPK, LC3, or β-actin levels were detected by an immunoblotting analysis. The ratios of pAMPK to total AMPK (B) and LC3-II to β-actin (C) were calculated as shown in the representative blots. *, *P < *0.05; **, *P < *0.01. Download FIG S4, TIF file, 0.2 MB. Copyright © 2020 Chen et al. 2020 Chen et al. This content is distributed under the terms of the Creative Commons Attribution 4.0 International license.

Using an immunofluorescence assay (IFA), we demonstrated an elevated endogenous LC3 punctum formation in THP-1 cells treated with TWEAK (Fig. 3B). The autophagic activity was further examined using THP-1 cells stably expressing the red fluorescent protein (RFP)-green fluorescent protein (GFP)-LC3 fusion protein. The results showed that TWEAK induced a redistribution of the RFP-GFP-LC3 fusion protein from a diffuse to a punctate pattern (Fig. 3C). Additionally, numbers of both yellow and red puncta increased in cells with TWEAK treatment (*P < *0.01), suggesting that TWEAK induces autophagy with an increased autophagic flux. Autophagosome maturation and acidification are crucial host processes against mycobacterial infection. To further examine whether TWEAK can induce autophagosome acidification, THP-1 cells were treated with TWEAK or IFN-γ (10 ng/ml), an inducer of autophagosome acidification (17), for 24 h and then incubated with a specific fluorescent marker for autophagosome acidification (LysoTracker Green). A significantly increased autophagosome acidification ratio was detected in cells with TWEAK treatment compared to that of control cells (33.7 ± 4.0 versus 7.2 ± 1.5%, *P < *0.005) (Fig. S5). The TWEAK effect on enhanced autophagosome acidification was counteracted by treatment with the classical inhibitor of autophagosome formation, 3-MA (8.7 ± 0.8%, *P < *0.005).

10.1128/mBio.03045-19.8 FIG S5 TWEAK induces autophagosome formation. THP-1 cell-derived macrophages were treated with the indicated reagent for 24 h and then stained with LysoTracker Green (LT; 2 μM). Autophagosome acidification was detected by confocal microscopy (A) and quantified (B). All experiments were performed in triplicate, and data are presented as means ± SEMs. The scale bar in the IFA image represents 5 μm. ***, *P < *0.005. Download FIG S5, TIF file, 0.4 MB. Copyright © 2020 Chen et al. 2020 Chen et al. This content is distributed under the terms of the Creative Commons Attribution 4.0 International license.

### TWEAK promotes mycobacterial phagosome maturation to inhibit mycobacterial survival.

Gutierrez et al. (1) demonstrated that autophagy promotes autophagosome maturation to inhibit mycobacterial survival in macrophages. To understand whether TWEAK-induced autophagy affects the formation of mycobacterial autophagosomes, THP-1 cells were treated with TWEAK, rapamycin, or starvation (a conventional inducer of autophagy) for 24 h before infection. Cells were infected with Texas Red-labeled BCG or H37Rv at an MOI of 10, and the formation of autophagosomes containing BCG or H37Rv was examined using confocal microscopy. Our results showed an elevated autophagosome formation, reflected by colocalization of BCG or H37Rv with LC3 (BCG cells, 68.9% ± 1.4% versus 21.4% ± 2.5%, *P < *0.005 [Fig. 3D]; H37Rv cells, 25.8% ± 1.9% versus 19.3% ± 3.1%, *P < *0.05 [Fig. S6A]), and autophagosome maturation (68.8% ± 4.2% versus 36.3% ± 5.8%, *P < *0.005 [Fig. S6C and Fig. S6D]) in TWEAK-treated cells compared with those in control cells. Additionally, the effect of TWEAK on mycobacterial autophagosome formation was abrogated in cells treated with an autophagy inhibitor (3-MA). These findings suggest that TWEAK-induced autophagy promotes mycobacterial autophagosome formation and maturation.

10.1128/mBio.03045-19.9 FIG S6 TWEAK promotes mycobacterial autophagosome maturation. (A) THP-1 cell-derived macrophages were treated with the indicated reagent for 24 h and then infected with Texas Red-labeled M. tuberculosis H37Rv for 2 h and stained with LC3 antibody. LC3 puncta (green) and H37Rv (red) were detected by confocal microscopy and quantified. (B) THP-1 cell-derived macrophages were transiently transfected with pSELECT-GFP-LC3. After 16 h, cells were treated with the indicated reagent for 24 h and then infected with Texas Red-labeled BCG for 2 h. The colocalization of BCG with GFP-LC3 was detected by confocal microscopy and quantified. (C and D) THP-1 cell-derived macrophages were treated with the indicated reagent for 24 h and then infected with Texas Red-labeled BCG for 2 h and stained with LysoTracker Green (LT; 2 μM) (C) or the specific autophagic vacuole fluorescent dye monodansylcadaverine (MDC; 50 mM) (D). The colocalization of BCG with MDC-positive autophagic vacuoles was detected by confocal microscopy and quantified. All experiments were performed in triplicate, and data are presented as means ± SEMs. The scale bar in the IFA image represents 5 μm. *, *P < *0.05; **, *P < *0.01; ***, *P < *0.005. Download FIG S6, TIF file, 1.7 MB. Copyright © 2020 Chen et al. 2020 Chen et al. This content is distributed under the terms of the Creative Commons Attribution 4.0 International license.

To elucidate the effect of TWEAK on autophagy during mycobacterial infection, THP-1 cell-derived macrophages were transfected with TWEAK small interfering RNA (siRNA) (siTWEAK) to knock down TWEAK expression (Fig. 3E), and then cells were infected with BCG. A significantly decreased TWEAK expression was observed in cells transfected with siTWEAK, indicating the efficiency of knockdown (Fig. 3F). An approximately 4.30-fold-greater expression of LC3-II was detected in THP-1 cells at 24 h after BCG infection than prior to infection (1.00-fold ± 0.00-fold [control cells] versus 4.30-fold ± 0.26-fold [BCG-infected cells], *P < *0.005) (Fig. 3G); however, a reduced LC3-II expression was noted in TWEAK knockdown cells (3.00-fold ± 0.36-fold, *P < *0.05) compared with that of control knockdown cells. In addition, an approximately 1.5-fold-higher survival of mycobacteria was detected in TWEAK knockdown cells than in control knockdown cells (BCG cells, 1.47-fold ± 0.01-fold; H37Rv cells, 1.77-fold ± 0.09-fold, *P < *0.01 [both]) (Fig. 3H).

### TNF-α stimulation induced miR-889 upregulation in granulomas.

**FIG 4:**
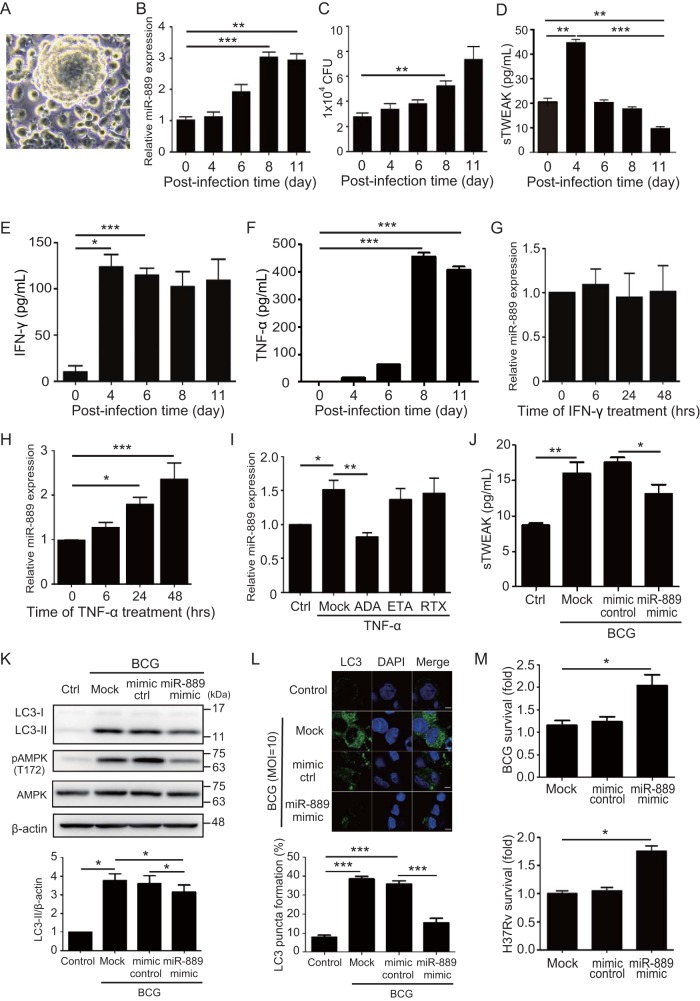
TNF-α stimulates miR-889 upregulation, and miR-889 inhibits mycobacterium-induced autophagy to maintain mycobacterial survival. (A) *In vitro* tuberculosis granuloma-like structures are formed by M. bovis BCG infection of human PBMCs. (B) The kinetics of miR-889 expression in granuloma-like structures with BCG infection was measured using QRT-PCR. (C) Mycobacterial viability was examined using a CFU assay. (D to F) The kinetics of secretory TWEAK (D), IFN-γ (E), and TNF-α (F) levels from granuloma-like structures was measured using ELISA. (G and H) Human PBMCs were treated with IFN-γ (10 ng/ml) (G) or TNF-α (5 ng/ml) (H), and the dynamics of miR-889 expression was measured using QRT-PCR. (I) Human PBMCs were cultured with TNF-α (5 ng/ml) for 24 h, and then an individual biologic (10 μg/ml) was added. At 24 h posttreatment, miR-889 expression was measured using QRT-PCR. ADA, adalimumab; ETA, etanercept; RTX, rituximab. (J to M) MicroRNA-889 inhibits mycobacterium-induced autophagy to maintain mycobacterial survival. THP-1 cell-derived macrophages were infected with BCG (MOI = 10) in the presence of the miR-889 mimic or a mimic control (30 nM) for 24 h. (J) Comparison of secretory TWEAK levels in miR-889 mimic-transfected cells, control-transfected cells, or nontransfected cells (mock). (K) The LC3 or β-actin levels were detected by an immunoblotting analysis (upper panel), and the ratios of LC3-II to β-actin were calculated (lower panel). (L) Endogenous LC3 was stained with LC3 antibody (green), and LC3 puncta were detected by confocal microscopy and quantified. (M) Mycobacterial survival was determined by a CFU assay at 24 h postinfection. (Upper panel) M. bovis BCG; (lower panel) M. tuberculosis H37Rv. All experiments were performed in triplicate, and data are presented as means ± SEMs. The scale bar in the IFA image represents 5 μm. *, *P < *0.05; **, *P < *0.01; ***, *P < *0.005.

Next, we dissected the role of miR-889 in latent mycobacterial infection by using an *in vitro* human TB granuloma model. We observed a granuloma-like structure formation at 8 days postinfection (Fig. 4A) with an increased expression of miR-889 (Fig. 4B), which paralleled the growth of mycobacteria (Fig. 4C). In contrast to miR-889 levels, the TWEAK levels declined from 4 days postinfection (Fig. 4D). Given that IFN-γ and TNF-α are critical for granuloma formation (18), we observed that IFN-γ levels peaked at day 4 postinfection and remained constant (Fig. 4E). Additionally, we similarly revealed significantly higher TNF-α levels at the time of granuloma formation than at baseline (456.1 ± 12.9 pg/ml versus 0.9 ± 0.9 pg/ml, *P < *0.005) (Fig. 4F), which parallels the upregulation of miR-889 (Fig. 4B). To investigate the association between IFN-γ or TNF-α and miR-889 expression, human PBMCs were treated with IFN-γ (10 ng/ml) or TNF-α (5 ng/ml) and the miR-889 expression was measured using QRT-PCR. As shown in Fig. 4G, there was no dramatic difference in miR-889 expression after IFN-γ stimulation. However, TNF-α induced upregulation of miR-889 expression in a time-dependent manner (Fig. 4H). To confirm this observation, human PBMCs were treated with TNF-α for 24 h, and then a TNF-α inhibitor (adalimumab or etanercept) was added. The results revealed that TNF-α-induced miR-889 upregulation was inhibited after the addition of adalimumab, a monoclonal antibody targeting soluble TNF-α (1.51-fold ± 0.14-fold versus 0.82-fold ± 0.07-fold, *P < *0.01) (Fig. 4I).

### miR-889 inhibits mycobacterium-induced autophagy to maintain mycobacterial survival by targeting TWEAK.

To dissect the biological function of miR-889 in mycobacterial infection, THP-1 cells were infected with BCG in the presence of miR-889 to mimic transfection, and then TWEAK expression levels were examined and quantified using an IFA and enzyme-linked immunosorbent assay (ELISA), respectively. As shown in Fig. S7A, a markedly higher expression of miR-889 in THP-1 cells occurred after transfection with the miR-889 mimic, indicating its effective transfection. The results of both the IFA (Fig. S7B) and the ELISA (Fig. 4J) showed increased TWEAK levels in the cells with BCG infection (16.1 ± 1.8 pg/ml) compared with those without infection (9.0 ± 0.1 pg/ml, *P < *0.01). Significantly lower levels of TWEAK were observed in miR-889-overexpressing cells after 24 h of infection (13.2 ± 1.2 pg/ml) than with mimic control-expressing cells (17.8 ± 0.7 pg/ml, *P < *0.05) or nontransfected cells (16.1 ± 1.8 pg/ml).

10.1128/mBio.03045-19.10 FIG S7 MicroRNA-889 inhibits mycobacterium-induced autophagosome acidification. THP-1 cell-derived macrophages were infected with BCG (MOI = 10) in the presence of an miR-889 mimic or a mimic control (30 nM) for 24 h. Comparison of miR-889 (A) and intracellular TWEAK (B) expression levels in miR-889 mimic-transfected, control-transfected, or nontransfected cells (mock). (C) Autophagosome acidification was stained with LysoTracker Green, detected by confocal microscopy, and quantified. All experiments were performed in triplicate, and data are presented as means ± SEMs. The scale bar in the IFA image represents 5 μm. **, *P < *0.01; ***, *P < *0.005. Download FIG S7, TIF file, 0.8 MB. Copyright © 2020 Chen et al. 2020 Chen et al. This content is distributed under the terms of the Creative Commons Attribution 4.0 International license.

To understand whether miR-889 regulates autophagy during mycobacterial infection, LC3 conversion levels were measured using immunoblotting (Fig. 4K). In contrast with results for mimic control-expressing cells, our results revealed declined levels of LC3-II expression in miR-889-overexpressing cells, which had smaller amounts of TWEAK simultaneously. By using an IFA to detect the endogenous LC3 punctum formation (Fig. 4L), the results showed that miR-889 inhibited BCG-induced LC3-II punctum formation compared with that induced by the mimic control (15.4% ± 2.4% versus 35.9% ± 1.6%, *P < *0.005). We further examined the effect of miR-889 on mycobacterial autophagosome maturation and acidification (Fig. S7C). A significant decrease in the autophagosome acidification ratio was observed in miR-889-overexpressing cells with BCG infection (8.3 ± 1.5%) compared to that of mimic control transfection cells (23.0% ± 2.1%, *P < *0.01) or nontransfected cells (21.7% ± 2.3%, *P < *0.01). Finally, to investigate whether miR-889 affected mycobacterial survival in macrophages, the viability of mycobacteria in miR-889-overexpressing cells was measured using a CFU assay. As shown in Fig. 4M, approximately 2-fold-increased mycobacterial survival was detected in miR-889-overexpressing cells than in control cells (BCG cells, 2.04-fold ± 0.02-fold; H37Rv cells, 1.75-fold ± 0.09-fold, *P < *0.05 [both]).

### Adalimumab affected miR-889 and TWEAK expression in BCG-induced granulomas.

**FIG 5:**
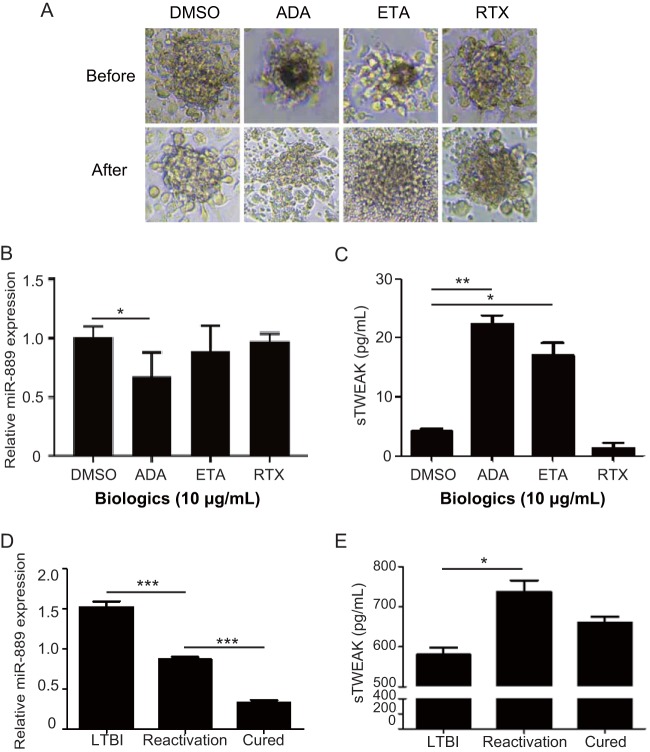
Significantly increased TWEAK expression in patients with LTBI reactivation. Human PBMCs were infected with BCG at an MOI of 0.1. At 8 days postinfection, the granuloma-like structure was treated with an individual biologic (10 μg/ml). (A to C) At 24 h posttreatment, the morphologies of the granulomas (A), miR-889 expression (B), and secretory TWEAK levels (C) were observed and measured. (D and E) Dynamics of miR-889 expression (D) and secretory TWEAK levels (E) in one RA patient with LTBI during an adalimumab therapy period. Data are presented as means ± SEMs. *, *P < *0.05; **, *P < *0.01; ***, *P < *0.005. ADA, adalimumab; ETA, etanercept; RTX, rituximab.

We further analyzed the miR-889 and TWEAK expression in biologic-related LTBI reactivation. At 8 days postinfection, the granuloma-like structure was constructed, and cells were treated with biologics. At 24 h posttreatment, a destructured granuloma was observed in cells treated with adalimumab (Fig. 5A) compared with that in dimethyl sulfoxide (DMSO)- or rituximab-treated cells. Additionally, decreased miR-889 expression (0.68-fold ± 0.21-fold, *P < *0.05) (Fig. 5B) and increased TWEAK levels (22.4 ± 1.4 pg/ml versus 4.2 ± 0.4 pg/ml, *P < *0.01) (Fig. 5C) were detected simultaneously. Besides adalimumab levels, TWEAK levels were significantly increased in cells treated with another TNF-α inhibitor (etanercept at 17.0 ± 2.1 pg/ml, *P < *0.05), but no significant change was observed in cells treated with rituximab, a B-cell depletion biologic).

### Significantly increased TWEAK expression in patients with LTBI reactivation.

To evaluate whether miR-889 or TWEAK expression reflects the different statuses of TB infection, we examined the dynamic change of miR-889 (Fig. 5D) and TWEAK (Fig. 5E) in the sera of one LTBI patient receiving adalimumab therapy. The examination revealed an elevation of miR-889 expression (1.52-fold ± 0.07-fold) and a decrease of TWEAK levels (580.0 ± 17.9 pg/ml) before TNF-α inhibitor therapy. However, TWEAK levels increased at the time of LTBI reactivation (736.9 ± 28.4 pg/ml, *P < *0.01 versus baseline) (Fig. 5E), and active TB disease developed. After anti-TB therapy was completed, both the miR-889 and TWEAK levels returned to baseline values.

## DISCUSSION

The immunopathogenesis of M. tuberculosis is complex (19) and is attributed mainly to immune-evading strategies that allow the pathogen to remain dormant after primary infection, persisting in the host and reactivating its pathogenicity under favorable conditions (20). It is estimated that nearly one-quarter of the world’s population has been latently infected with M. tuberculosis, and this population is an important reservoir for disease reactivation. LTBI is an equilibrium status between the host and mycobacterium during infection. Host responses in controlling the latent infection may include macrophage activation, maintenance of granuloma structure, and expression of IFN-γ, TNF-α, CD4 T cells, and CD8 T cells (21). In the present study, we revealed a novel mechanism of fine-tuned balance between mycobacteria and the host for granuloma formation and/or maintenance in patients with the LTBI status. Our results showed that miR-889 expression was significantly higher in RA patients with LTBI than in those without infection or in healthy controls, and its expression was positively correlated with released levels of IFN-γ in the QFT-G assay (*r* = 0.63, *P < *0.001). A significant decrease of miR-889 levels paralleled the prophylactic therapy for LTBI patients, indicating that miR-889 expression might be involved in M. tuberculosis infection, which is consistent with the conclusions of another study (22). Given that TWEAK acts as the target of miR-889, we found significantly lower TWEAK levels in miR-889-overexpressing cells and in LTBI patients who had high levels of expression of miR-889 than in other subjects. Moreover, TWEAK levels increased at the time of LTBI reactivation. These findings suggest that miR-889 and TWEAK may act as candidate biomarkers for LTBI and LTBI reactivation, respectively. Further large studies are required to confirm our data.

Autophagy is a well-conserved lysosomal degradation pathway that plays a key role in the innate defense mechanism against mycobacteria (23). Dormant M. tuberculosis can suppress autophagy and then survive within macrophages for an extended period, which is responsible for LTBI (24). Our results showed that a mycobacterial component (e.g., Ag85A, Ag85B) induced TWEAK upregulation. TWEAK, a member of the TNF superfamily, regulates several cellular responses, including proinflammatory activity, angiogenesis, and apoptosis (25). TWEAK balances TNF-α activity by repressing the production of proinflammatory cytokines in modulating the transition from innate to adaptive immunity (26). In the present study, we demonstrated that TWEAK induced autophagy and promoted autophagosome maturation against mycobacterial infection through activation of AMPK. AMPK is an essential metabolic regulator that plays an important role in the maintenance of energy balance in response to stress (27). Additionally, AMPK plays a crucial role in the initiation of autophagy with subsequent autophagosome formation and maturation (28). Activated AMPK induces the phosphorylation of the serine residues (e.g., Ser555) of ULK1, which is a mammalian autophagy-initiating kinase that plays a key role in starvation-induced autophagy (29). A previous study demonstrated that ULK1 was strongly associated with LTBI and may play a crucial role in the regulation of autophagy and mycobacterial replication (30). In addition to demonstrating autophagy, recent studies demonstrated that AMPK is involved in metabolic responses, fatty acid β-oxidation, and the control of pathological inflammation in macrophages during M. tuberculosis infection (31). We identified a novel role of TWEAK in AMPK activation in antimycobacterial autophagy. More, in-depth studies of the regulatory mechanism of TWEAK in AMPK-targeted effector networks in mycobacterial infection are needed to confirm our finding.

**FIG 6:**
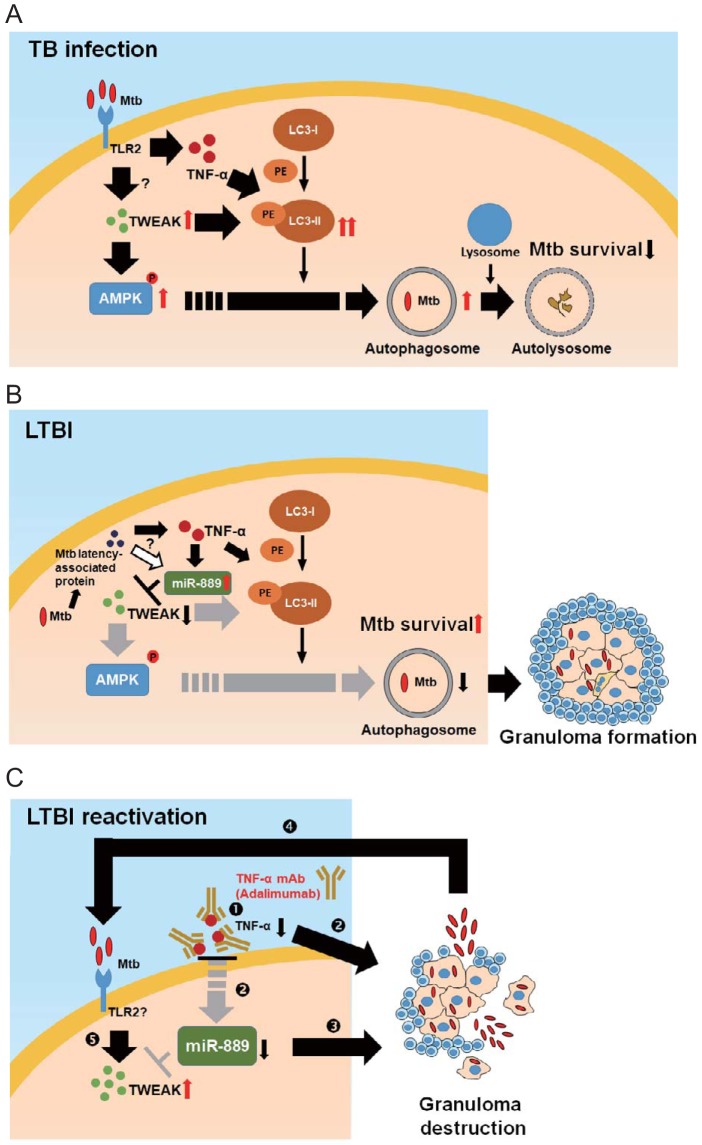
Proposed models for the biological roles of TWEAK and miR-889 in TB infection (A), LTBI (B), and LTBI reactivation (C), based on the results of this study. (A) In early infection, M. tuberculosis (Mtb) stimulates TWEAK expression. TWEAK induces autophagy through activation of AMPK against mycobacterial infection. PE, phycoerythrin. (B) In a patient with the LTBI status, TNF-α mediates granuloma structure formation and maintenance. Latency-associated proteins of M. tuberculosis may induce TNF-α and miR-889, thus inhibiting autophagy by targeting TWEAK and then repressing autophagosome maturation and maintaining mycobacterial survival. (C) Adalimumab neutralizes TNF-α and reduces its levels, and then it affects miR-889 expression and disrupts granuloma structure. The destructured granuloma causes mycobacteria to emerge from dormancy and develop into active TB (LTBI reactivation). Increased numbers of mycobacteria stimulate TWEAK expression. mAb, monoclonal antibody.

TB infection is highly dynamic and determined by the interaction between the host and the mycobacterium (32). Granuloma formation plays a crucial role in TB pathogenesis (33). The cellular factors that control granuloma formation and maintenance are multifaceted, involving a complex interplay between the host immune system and mycobacterium survival strategies (32). TNF-α is critical for granuloma formation (18). Previous studies demonstrated a fine-tuning of TNF-α production in the host during TB infection, which allowed mycobacterial persistence in granulomas without apparent disease (LTBI status) (18, 32–34). Agarwal et al. (35) validated that mycobacteria can use this host granulomatous response to continue infection. In this study, we found an increased expression of TWEAK during early mycobacterial infection, which induced autophagy and promoted mycobacterial autophagosome maturation through the activation of AMPK (Fig. 6A). Our results revealed that the role of TWEAK in mycobacterial infection is similar to that of TNF-α, which has been known to induce autophagy and phagosome maturation in macrophages (17, 36). Previous studies demonstrated that once the organism entered latent TB infection status, some latency-associated proteins of M. tuberculosis maintained mycobacterial survival in granulomas by stimulating the expression of TNF-α (37) or inhibiting autophagy (38). Our results showed that TNF-α inhibits TWEAK expression in macrophages through upregulating miR-889 expression to maintain granuloma formation (Fig. 6B), supporting the findings that TNF-α is responsible for granuloma formation and maintenance in subjects with LTBI (18). More studies are needed to dissect the regulatory mechanism between TNF-α and TWEAK, from TB infection to LTBI. Additionally, upregulated miR-889 inhibited mycobacterium-caused autophagy to maintain mycobacterial survival in granulomas, suggesting that miR-889 expression might be associated with latent M. tuberculosis infection. The association between miR-889 expression and latency-associated proteins of M. tuberculosis requires further studies to dissect.

Increasing evidence reveals that TNF-α inhibitors are associated with an elevated risk of LTBI reactivation (39, 40). Since TNF-α is involved in granuloma maintenance, neutralizing TNF-α tends to disrupt the granuloma structure, allowing mycobacteria to emerge from dormancy and develop into active TB (41). A previous study inferred that TNF-α inhibitors may reduce autophagy; then, the repressive effect on autophagy may be responsible for the increased TB risk (36). We revealed an upregulation of miR-889 by soluble TNF-α; adalimumab then reduced TNF-α levels and thus affected the expression of miR-889, resulting in granuloma destruction (Fig. 6C). Once granuloma destruction and LTBI reactivation occurred, TWEAK expression might have been enhanced by mycobacterium induction. Our findings were consistent with another mouse LTBI granuloma model showing increased TWEAK levels after treatment with TNF-neutralizing antibody (42). Additionally, significantly elevated TWEAK levels were observed in our adalimumab-treated patient exhibiting LTBI reactivation, which may be associated with increased mycobacterial stimulation. These observations suggest that TWEAK may be a potential biomarker for predicting LTBI reactivation during the period of biologic therapy.

This study had some limitations. First, it lacked LTBI subjects without rheumatic disease, and the number of healthy control subjects was small. Previous studies demonstrated that deregulation of the autophagic pathway and TWEAK/Fn14 signaling are involved in the pathogenesis of RA (43, 44), which may be risks of bias in this study. Additionally, the study was cross-sectional by design, and thus, the possibility that miRNA expression changed with therapeutic strategies cannot be excluded. However, we validated our observations by using an *in vitro* cell-based assay, suggesting that our results still provided valuable information.

The mycobacterium-containing granuloma represents a dynamic balance between host and pathogen (45). Modulation of host-specific immune responses can limit mycobacterial infection. TWEAK is a multifunctional cytokine and may be used as a therapeutic target for rheumatic diseases. The biologic agent that blocks TWEAK (BIIB023) has completed a phase II clinical trial in rheumatic-disease therapy (46, 47). Here, we revealed a relationship between TWEAK and activation of the autophagic machinery, which promotes antimycobacterial immunity. In-depth studies are required to demonstrate the effect of a TWEAK inhibitor on TB disease and LTBI reactivation. Additionally, we explored an effect of miR-889 on antimycobacterial autophagy in patients with LTBI and LTBI reactivation. Given that the guidelines recommend LTBI screening for patients prior to biologic treatment (48, 49), miR-889 and TWEAK may be diagnostic biomarkers of or therapeutic targets for LTBI or LTBI reactivation, respectively. Further studies are required to confirm and extend our findings for their clinical implications.

## MATERIALS AND METHODS

### Subjects.

This prospective study was conducted at a medical center from 2014 to 2018. The Institutional Review Board of Taichung Veterans General Hospital approved this study (CE13330B), and the written consent of all participants was obtained according to the Declaration of Helsinki. Detailed definitions of subjects are available in Text S1 in the supplemental material.

10.1128/mBio.03045-19.1 TEXT S1 Supplemental materials and methods. Download Text S1, DOCX file, 0.03 MB. Copyright © 2020 Chen et al. 2020 Chen et al. This content is distributed under the terms of the Creative Commons Attribution 4.0 International license.

### MicroRNA isolation.

Total RNAs were extracted using TRIzol reagent (Invitrogen, ThermoFisher Scientific, USA) and purified using an RNeasy MinElute cleanup kit (Qiagen, Germany), according to the manufacturer’s instructions. Purified RNAs were quantified at an optical density at 260 nm (OD_260_) and an OD_280_ using an ND-1000 spectrophotometer (NanoDrop Technology, USA), and isolated miRNAs were qualified by capillary gel electrophoresis using a Bioanalyzer 2100 (Agilent Technology, USA).

### QRT-PCR.

MicroRNA expression was measured and quantified using a TaqMan microRNA assay kit (Applied Biosystems, ThermoFisher Scientific, USA), according to the manufacturer’s protocol. QRT-PCRs were performed on the StepOnePlus real-time PCR system (Applied Biosystems, ThermoFisher Scientific, USA), using a standard protocol. Detailed protocols are available in Text S1 in the supplemental material.

### *In vitro* tuberculosis granuloma model and CFU assay.

For granuloma formation, freshly isolated peripheral blood mononuclear cells (PBMCs) were infected with the M. bovis BCG or M. tuberculosis H37Rv strain at a multiplicity of infection (MOI) of 0.1; RPMI medium containing 20% human serum was added, and the mixture was incubated at 37°C in an incubator containing 5% CO_2_ for up to 11 days (50). The medium and sera were replenished every 4 days. Mycobacterial growth was determined using CFU assays. Briefly, infected granuloma-like structures were lysed at different time periods ranging from 4 to 11 days postinfection. Lysates were serially diluted and plated on Middlebrook 7H11 agar plates and then incubated at 37°C to determine the number of CFU at 14 days.

### Immunoblotting.

The cells with different treatments were lysed in RIPA buffer (25 mM Tris-HCl, pH 7.6, 150 mM NaCl, 1% NP-40, 1% sodium deoxycholate, and 0.1% sodium dodecyl sulfate [SDS]) containing a protease inhibitor cocktail (Complete, Roche, Germany). Twenty micrograms of total protein from exosome lysate was loaded and separated on a standard SDS-polyacrylamide gel electrophoresis (PAGE) gel and transferred to a polyvinylidene difluoride (PVDF) membrane (Millipore, USA). The membranes were incubated with primary antibodies, followed by peroxidase-conjugated secondary antibodies. The results were detected using a charge-coupled device (CCD) camera-based imager (GE Healthcare Life Sciences, USA) after membrane incubation with enhanced-chemiluminescence (ECL) substrates (Millipore, USA).

### Immunofluorescence assay.

THP-1 cell-derived macrophages with individual treatment were fixed with 4% paraformaldehyde at room temperature for 10 min and then washed three times with phosphate-buffered saline (PBS). Cells were permeabilized in PBS containing 1% bovine serum albumin (BSA) and 0.2% saponin and then blocked for 1 h in PBS containing 2% BSA. Cells were then incubated with the primary antibodies, followed by a secondary antibody. Coverslips were mounted onto glass slides with DAPI (4′,6-diamidino-2-phenylindole)-containing SlowFade mounting medium (ThermoFisher Scientific, USA), and images were observed and recorded on an Olympus FV1000 laser-scanning confocal microscope. Images were analyzed by using FV10-ASW version 4.2 software. For quantification of the cells showing LC3-positive vesicles, approximately 50 cells were counted, and the cells with more than 20 LC3-labeled puncta were labeled as having formed an autophagosome.

### Statistical analysis.

An unpaired, two-tailed Student *t* test was used for between-group comparisons. A one-way analysis of variance (ANOVA) with the *post hoc* Bonferroni test was used for multiple comparisons. The correlation coefficient was calculated using Spearman’s correlation test. *P* values of <0.05 were statistically significant, and tests were performed by using GraphPad Prism 7.
