# Supplementary material for: MicroRNA-889 Inhibits Autophagy To Maintain Mycobacterial Survival in Patients with Latent Tuberculosis Infection by Targeting TWEAK
Source: mBio. 2020 Jan 28;11(1):e03045-19. doi: 10.1128/mBio.03045-19 (PMC6989109; doi:10.1128/mBio.03045-19)
Supplement: TABLE S1 [file mBio.03045-19-st001.docx]

**Table S1** Demographic data and laboratory findings of rheumatoid arthritis (RA) patients with latent tuberculosis infection (LTBI), nontuberculous mycobacteria (NTM) infection, and without infection.

|  | Mycobacterial infection | | |
| --- | --- | --- | --- |
|  | LTBI | NTM | Without |
|  |  | infection | infection |
|  | (n=35) | (n=12) | (n=50) |
| Age at study entry (years) | 60.8±13.2 | 62.1±10.7 | 61.3±10.2 |
| Gender (female, %) | 26 (74.3) | 8 (66.7) | 38 (76.0) |
| Disease duration (years) | 9.5±5.2 | 9.6±4.5 | 9.8±4.8 |
| Comorbidity |  |  |  |
| diabetes mellitus | 5 (14.3) | 2 (16.7) | 8 (16.0) |
| chronic kidney disease | 5 (14.3) | 3 (25.0) | 7 (14.0) |
| RF (positive, %) | 27 (77.1) | 5 (41.6) | 36 (72.0) |
| Anti-CCP antibody (positive, %) | 31 (88.6) | 8 (66.7) | 35 (70.0) |
| DAS28 at study entry | 3.9±1.3 | 3.8±1.7 | 3.7±1.2 |
| ESR (mm/h) | 28.5±7.8 | 11.5±3.5 | 20.4±8.9 |
| CRP (mg/dL) | 0.95±1.45 | 1.14±1.15 | 0.98±1.42 |
| Daily prednisolone dose (mg) | 4.3±2.5 | 4.7±1.7 | 4.1±1.8 |
| Methotrexate (%) | 21 (60.0) | 4 (33.3) | 26 (52.0) |
| Hydroxychloroquine (%) | 19 (54.3) | 7 (58.3) | 18 (36.0) |
| Sulfasalazine (%) | 7 (20.0) | 2 (16.7) | 8 (16.0) |
| Biologics therapy (%) | 28 (80.0) | 9 (75.0) | 37 (74.0) |
| Adalimumab | 12 (34.3) | 3 (25.0) | 12 (24.0) |
| Etanercept | 8 (22.9) | 4 (33.3) | 9 (18.0) |
| Rituximab | 7 (20.0) | 2 (16.7) | 9 (18.0) |
| Abatacept | 0 (0) | 0 (0) | 4 (8.0) |
| Tocilizumab | 1 (2.9) | 0 (0) | 3 (6.0) |

*Values are mean±SD or the number (%) of patients.

RF, rheumatoid factor; CCP, cyclic citrullinated peptide; DAS28, disease activity score for 28-joints; ESR, erythrocyte sedimentation rate; CRP, C reactive protein.
