# Supplementary material for: MicroRNA-889 Inhibits Autophagy To Maintain Mycobacterial Survival in Patients with Latent Tuberculosis Infection by Targeting TWEAK
Source: mBio. 2020 Jan 28;11(1):e03045-19. doi: 10.1128/mBio.03045-19 (PMC6989109; doi:10.1128/mBio.03045-19)
Supplement: TABLE S2 [file mBio.03045-19-st002.docx]

**Table S2** Differentially expressed miRNAs in PBMCs from RA patients with LTBI compared with those without infection, identified by miRNA NGS analysis*

| Up-regulated miRNAs | Fold change^§^  (median value) | Down-regulated miRNAs | Fold change^§^  (median value) |
| --- | --- | --- | --- |
| hsa-miR-889 | 4.02 | hsa-miR-9-5p | 0.32 |
| hsa-miR-193a | 3.03 | hsa-miR-4676 | 0.30 |
| hsa-miR-21 | 2.22 | hsa-miR-1229 | 0.20 |
| hsa-miR-539-3p | 2.40 | hsa-miR-3657 | 0.32 |
| hsa-miR-941 | 2.14 | hsa-miR-634 | 0.28 |
| hsa-miR-485-3p | 2.32 | hsa-miR-450a | 0.28 |
|  |  | hsa-miR-7e-3p | 0.31 |
|  |  | hsa-miR-337 | 0.20 |
|  |  | hsa-miR-125a-3p | 0.27 |
|  |  | hsa-miR-766-5p | 0.24 |
|  |  | hsa-miR-32-3p | 0.16 |

*miRNA, microRNA; PBMCs, peripheral blood mononuclear cells; RA, rheumatoid arthritis; LTBI, latent tuberculosis infection; NGS, next generation sequencing.

^§^Fold change: if the number >2.00 or <0.33, the difference is considered significant.
