# Supplementary figures and images for: MicroRNA-889 Inhibits Autophagy To Maintain Mycobacterial Survival in Patients with Latent Tuberculosis Infection by Targeting TWEAK
Source: mBio. 2020 Jan 28;11(1):e03045-19. doi: 10.1128/mBio.03045-19 (PMC6989109; doi:10.1128/mBio.03045-19)

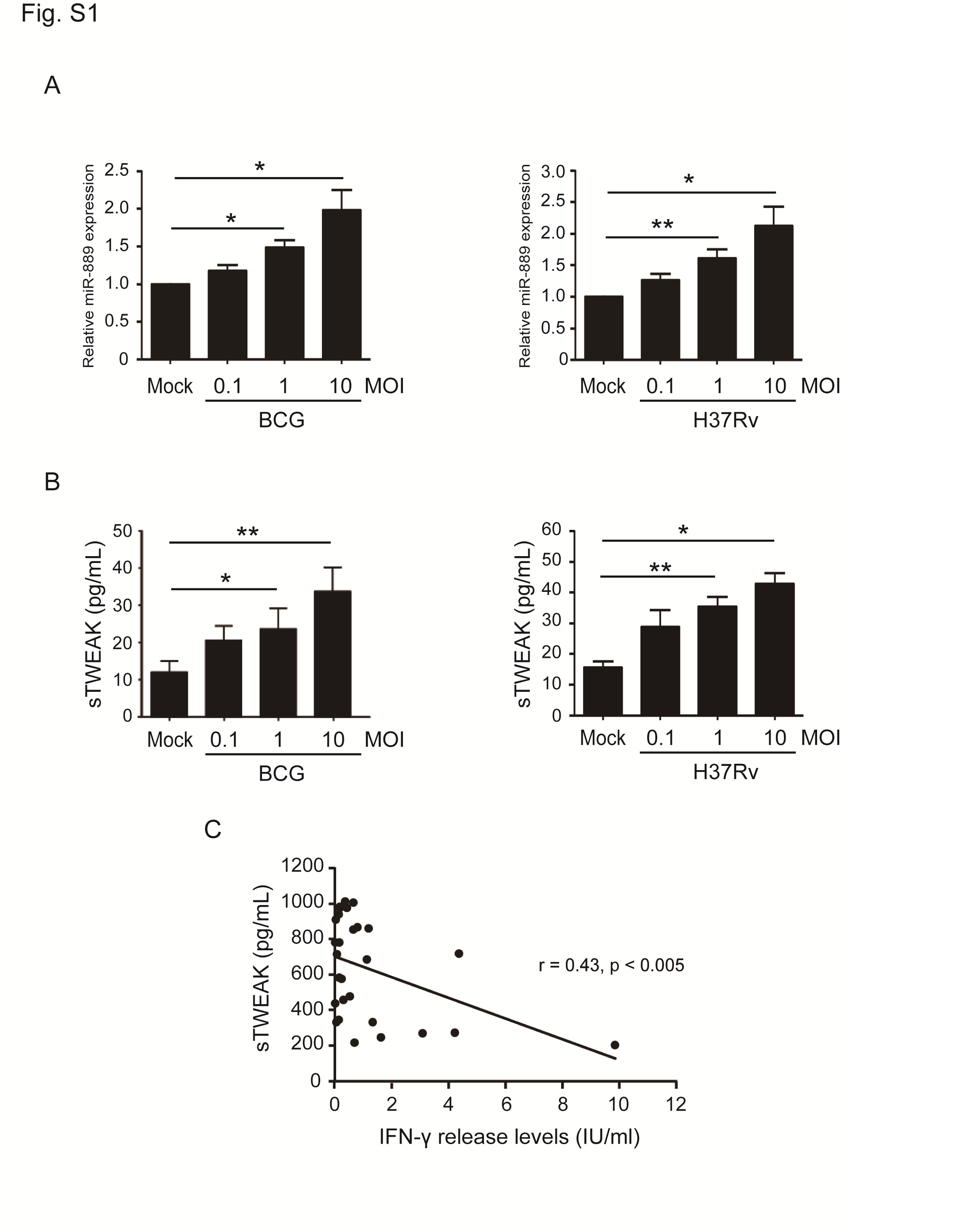

Supplement: FIG S1 [file mBio.03045-19-sf001.tif]

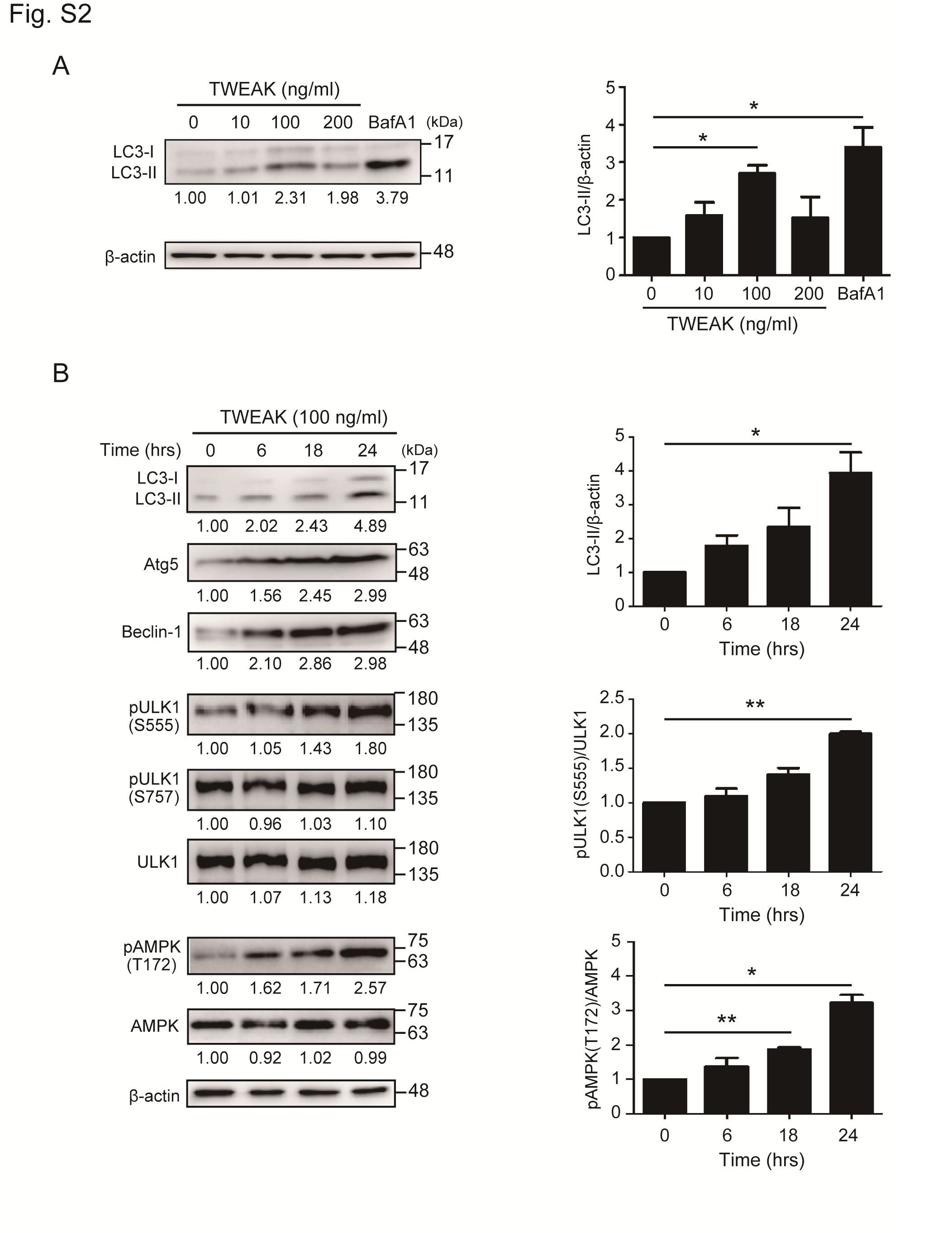

Supplement: FIG S2 [file mBio.03045-19-sf002.tif]

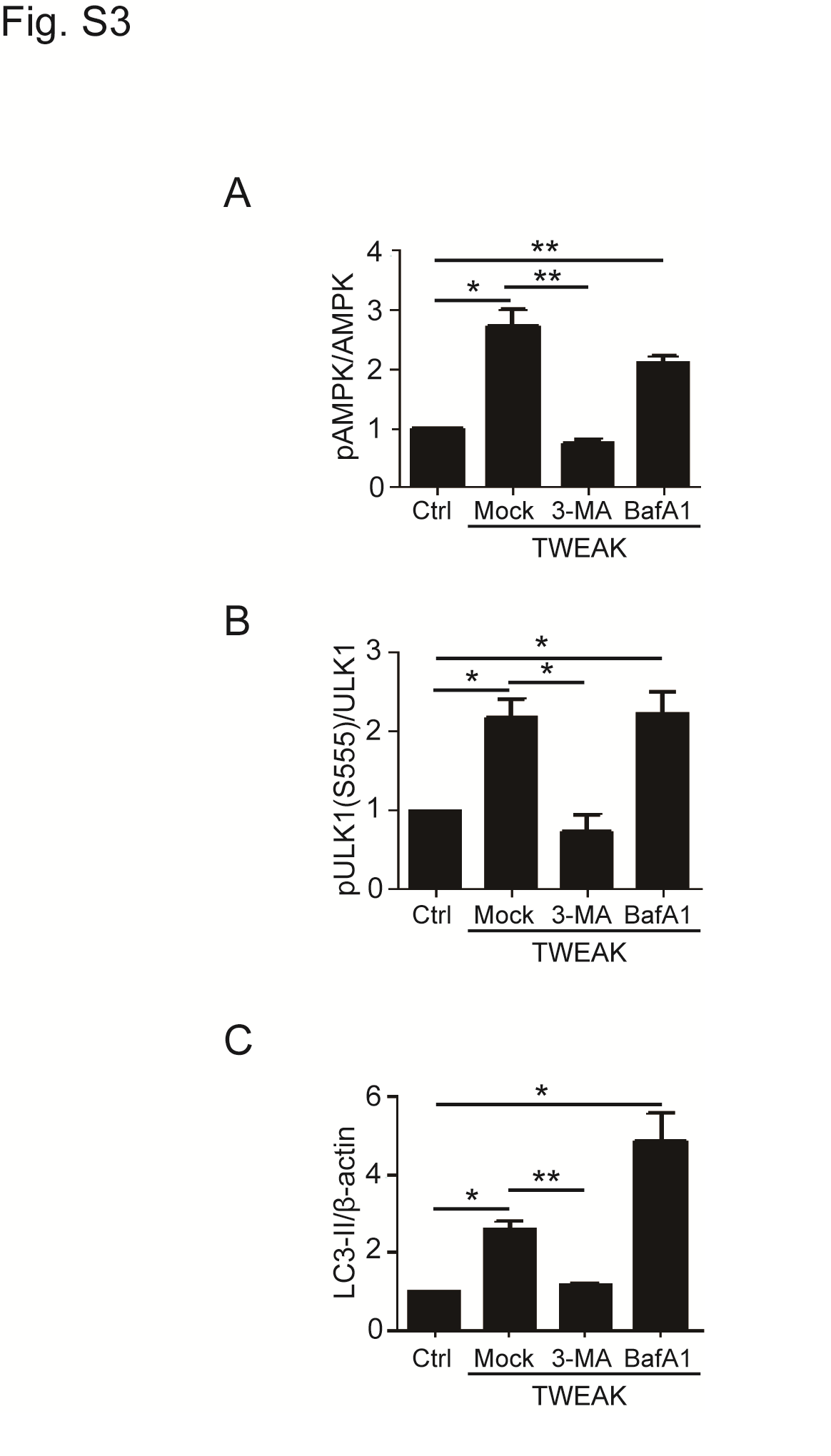

Supplement: FIG S3 [file mBio.03045-19-sf003.tif]

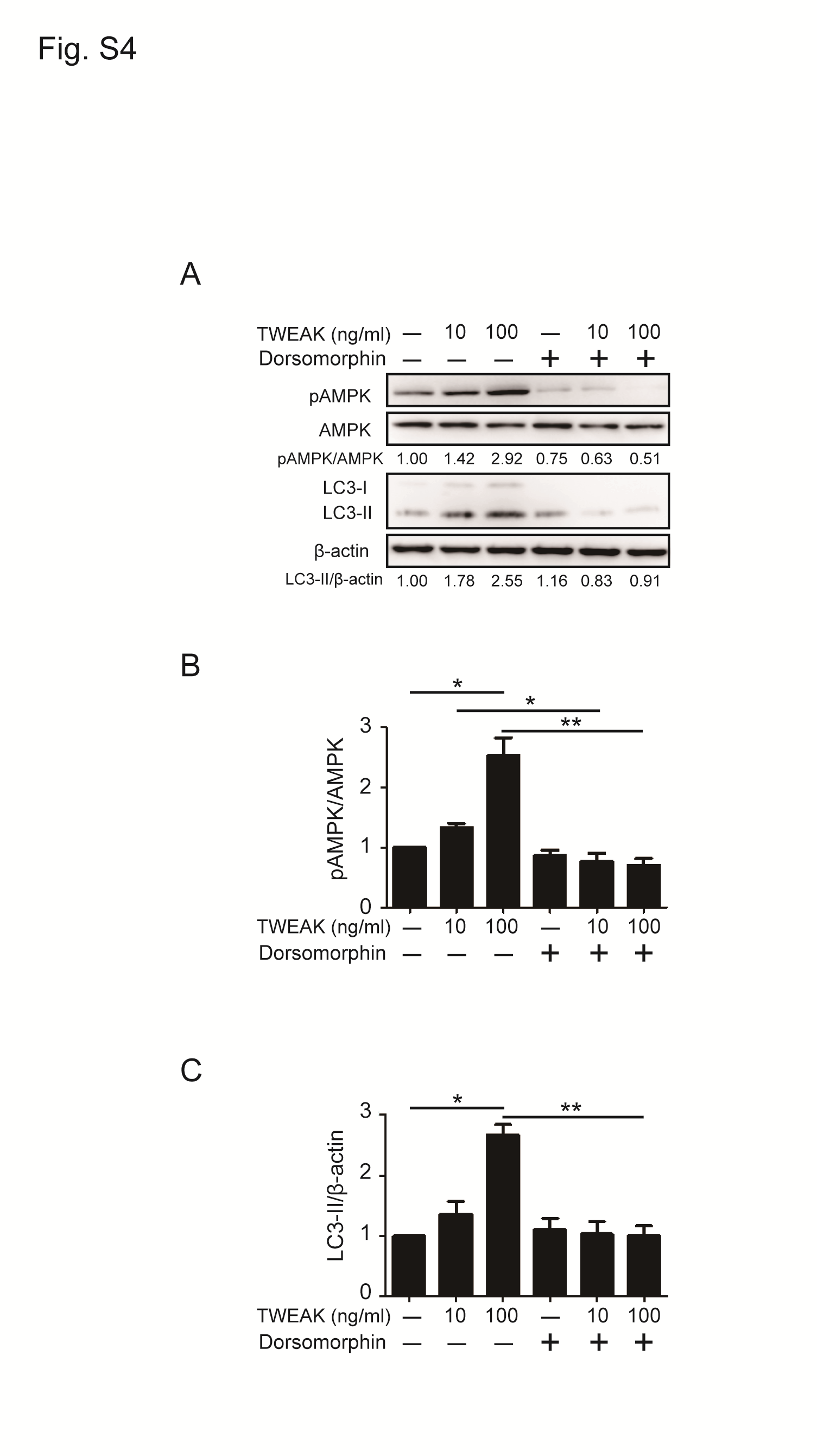

Supplement: FIG S4 [file mBio.03045-19-sf004.tif]

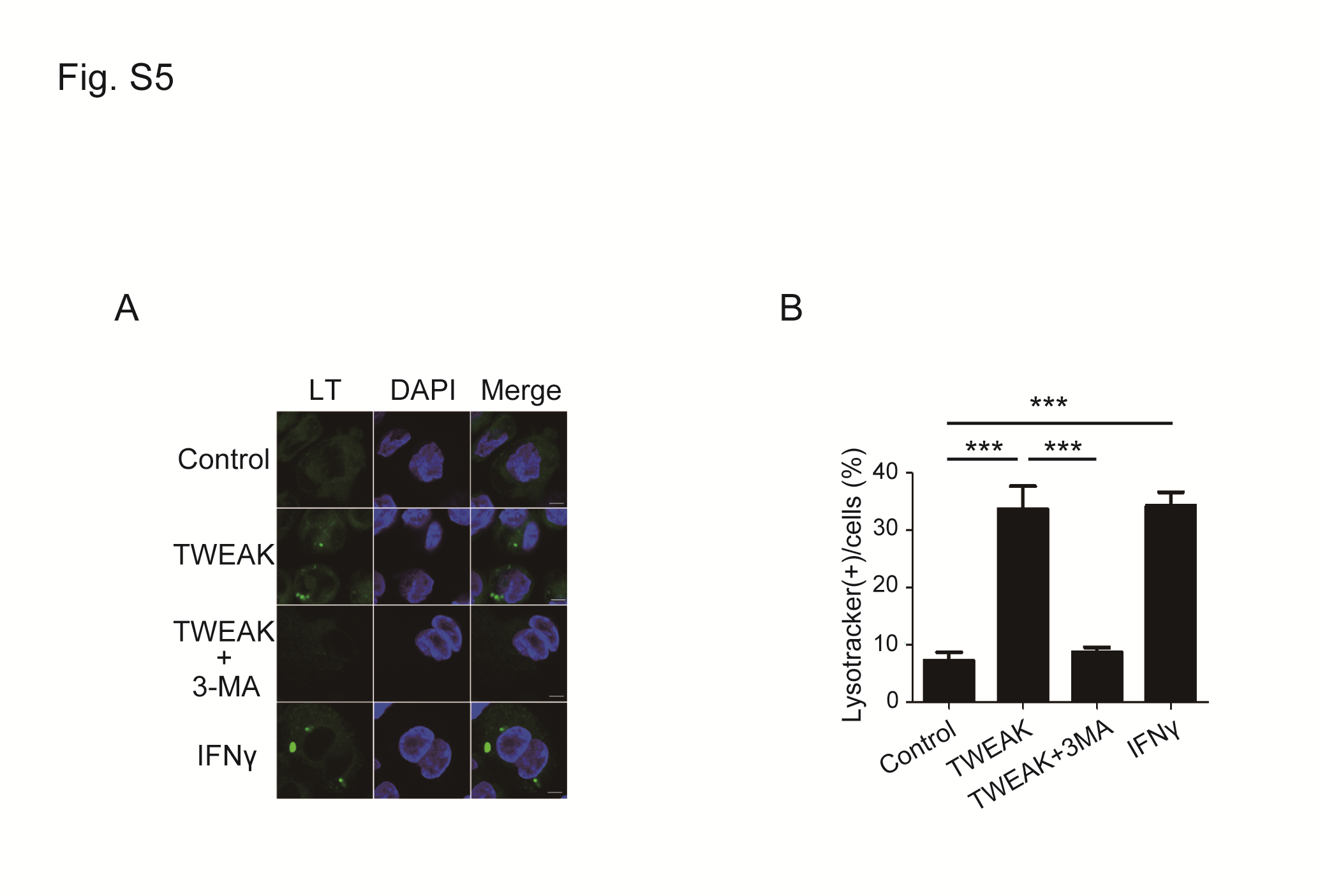

Supplement: FIG S5 [file mBio.03045-19-sf005.tif]

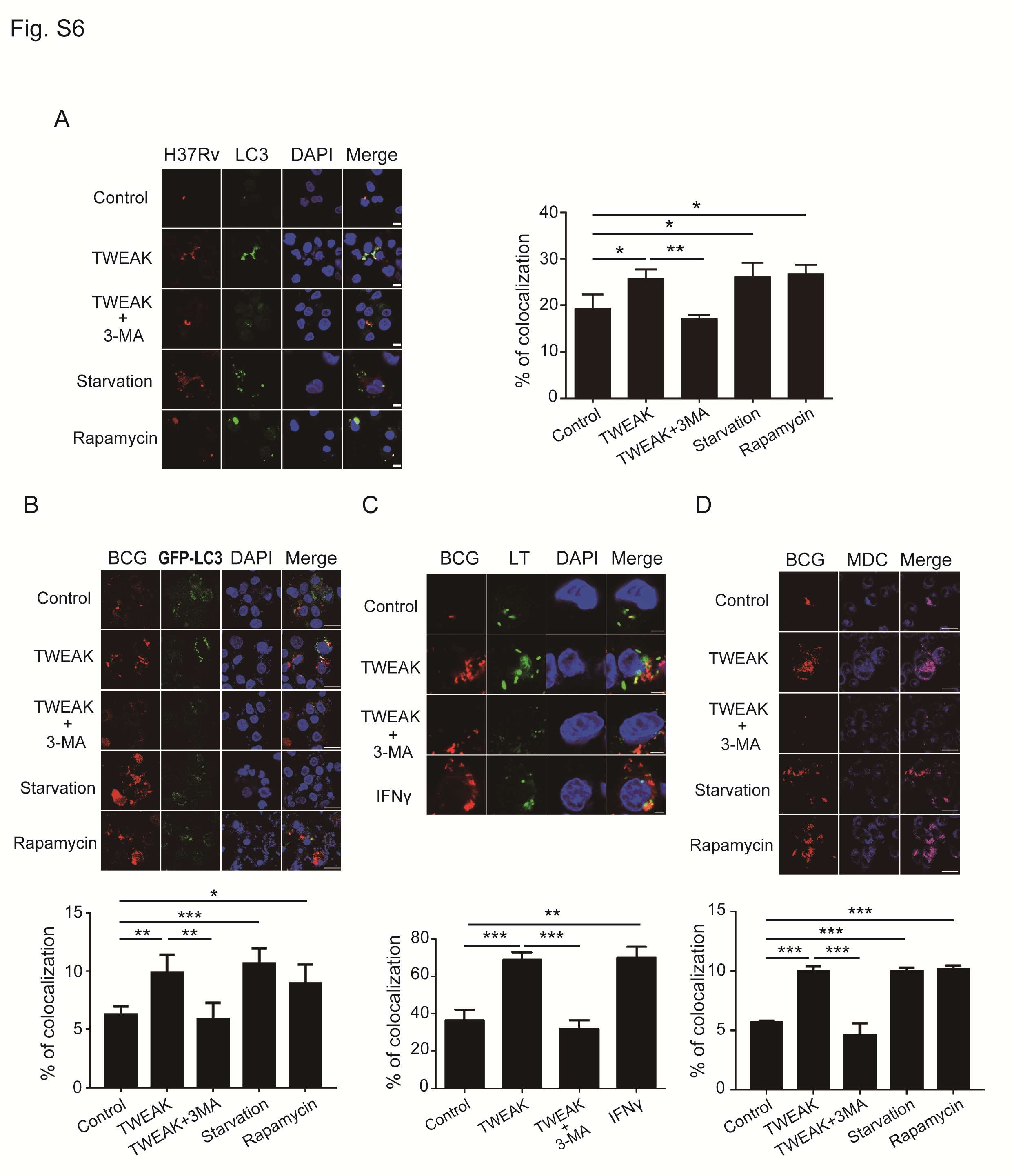

Supplement: FIG S6 [file mBio.03045-19-sf006.tif]

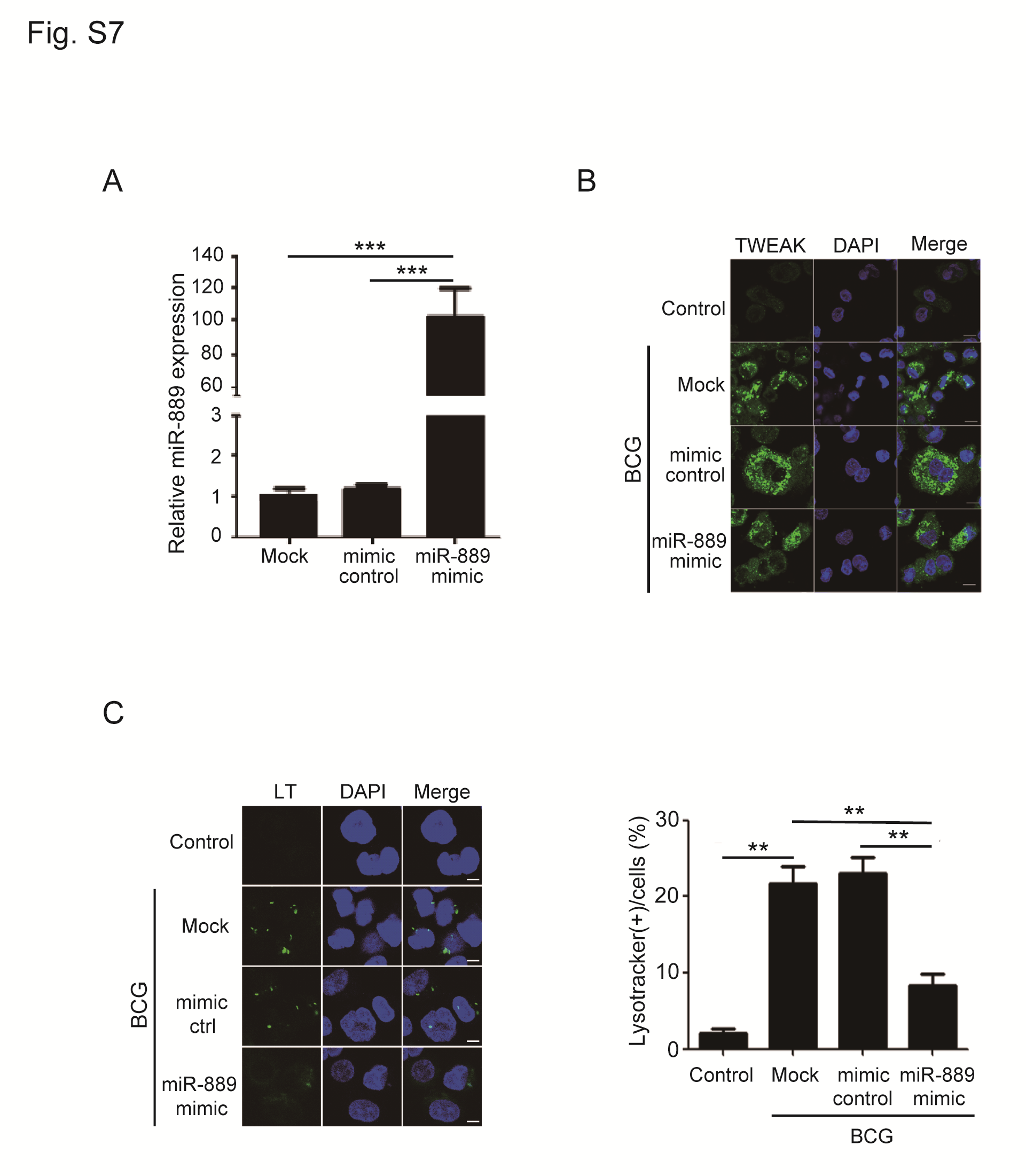

Supplement: FIG S7 [file mBio.03045-19-sf007.tif]
