# Supplementary material for: MicroRNA-889 Inhibits Autophagy To Maintain Mycobacterial Survival in Patients with Latent Tuberculosis Infection by Targeting TWEAK
Source: mBio. 2020 Jan 28;11(1):e03045-19. doi: 10.1128/mBio.03045-19 (PMC6989109; doi:10.1128/mBio.03045-19)
Supplement: TEXT S1 [file mBio.03045-19-s0001.docx]

**File S1.** Supplementary Materials and Methods

***Reagent or Resource***

| Reagent or Resource | Source | Identifier (Cat No.) |
| --- | --- | --- |
| Antibodies | | |
| Mouse anti-β-actin  antibody | Santa Cruz | sc-47778 |
| Rabbit anti-AMPKα  antibody | Cell Signaling Technology | #5832 |
| Rabbit anti-pAMPKα(T172) antibody | Cell Signaling Technology | #2535 |
| Rabbit anti-Atg5  antibody | Cell Signaling Technology | #12994 |
| Rabbit anti-Beclin-1 antibody | Cell Signaling Technology | #3738 |
| Rabbit anti-LC3B  antibody | Cell Signaling Technology | #2775 |
| Rabbit anti-pULK1(S555)  antibody | Cell Signaling Technology | #5869 |
| Rabbit anti-pULK1(S757)  antibody | Cell Signaling Technology | #14202 |
| Rabbit anti-ULK1  antibody | Cell Signaling Technology | #6439 |
| Rabbit anti-TWEAK antibody | Abcam | ab37170 |
| Alexa Fluor® 488 Rabbit anti-LC3B antibody | NOVUS | NB100-2220-AF488 |
| Alexa Fluor® 488 Goat anti-Rabbit IgG | ThermoFisher Scientific | A11008 |
| Anti-mouse IgG, HRP-linked antibody | Cell Signaling Technology | #7076 |
| Anti-rabbit IgG, HRP-linked antibody | Cell Signaling Technology | #7074 |
| Chemicals, Peptides, and Recombinant Proteins | | |
| 3-methyladenine (3-MA) | Sigma-Aldrich | M9281 |
| Bafilomycin A1 | Sigma-Aldrich | B1793 |
| Dorsomorphin | Sigma-Aldrich | P5499 |
| heat-killed *M. tuberculosis* (HKMT) | InvivoGen | tlrl-hkmt-5 |
| Interferon gamma (IFNγ) | PeproTech | 300-02 |
| Recombinant *Mycobacterium tuberculosis* Antigen 85A (Ag85A) | ProSpec | PRO-1076 |
| Recombinant *Mycobacterium tuberculosis* Antigen 85B (Ag85B) | ProSpec | PRO-589 |
| Lipofectamine RNAiMAX Transfection Reagent | ThermoFisher Scientific | 13778150 |
| Lipopolysaccharides (LPS) from *Escherichia coli* O55:B5 | Sigma-Aldrich | L2880 |
| LysoTracker™ Green | ThermoFisher Scientific | L7526 |
| monodansylcadaverine (MDC) | Sigma-Aldrich | 30432 |
| Peptidoglycan (PGN) | Sigma-Aldrich | 77140 |
| Phorbol myristate acetate (PMA) | Sigma-Aldrich | P1585 |
| Polyinosinic–polycytidylic acid sodium salt [poly (I:C)] | Sigma-Aldrich | P1530 |
| Rapamycin | Sigma-Aldrich | R0395 |
| Recombinant human TWEAK | PeproTech | 310-06 |
| Texas Red-X | ThermoFisher Scientific | T20175 |
| Trizol reagent | ThermoFisher Scientific | 15596018 |
| Tumor necrosis factor (TNF)-α | PeproTech | 300-01A |
| Zeocin | InvivoGen | ANT-ZN-1 |
| Cell lines | | |
| THP-1 Difluo^TM^ hLC3 cells | InvivoGen | THPDF-HLC3 |
| Others | | |
| has-miR-889-3p miRNA mimic | ThermoFisher Scientific | MC12495 |
| miRNA mimic Negative Control | ThermoFisher Scientific | 4464058 |
| has-miR-889-3p miRNA inhibitor | ThermoFisher Scientific | MH12495 |
| miRNA inhibitor Negative Control | ThermoFisher Scientific | 4464084 |
| has-miR-889-3p TaqMan MicroRNA Assays | ThermoFisher Scientific | 002202 |
| TWEAK TaqMan Gene Expression Assays | ThermoFisher Scientific | Hs00387540_g1 |
| Human TWEAK Instant ELISA kit | ThermoFisher Scientific | BMS2006INST |
| On TARGETplus SMARTpool siTWEAK | Dharmacon | L-010629-00-0005 |

*Subjects*

This prospective study was conducted at a medical center from 2014 to 2018. A total of 97 patients who fulfilled the 2010 revised criteria of the American College of Rheumatology for RA [1] and 23 healthy subjects were enrolled. RA activity was assessed according to the 28-joint disease activity score (DAS28) [2], and active status was defined as a DAS28 >3.2. RA patients were excluded if they had a clinically active TB or characteristic radiological findings suggesting healed pulmonary TB. Active TB was proven with a positive culture or pathological findings of a tissue biopsy. The definition of NTM disease was based on the American Thoracic Society and the Infectious Diseases Society of America guidelines [3]. The Institutional Review Board of Taichung Veterans General Hospital approved this study (CE13330B), and the written consent of all participants was obtained according to the Declaration of Helsinki.

***QuantiFERON-TB Gold (QFT-G) assay***

All patients were evaluated at baseline for TB infection using standardized interviews, physical examinations, and chest radiographs. The QFT-G assay was performed according to the manufacturer’s instructions (Cellestis Ltd., Victoria, Australia). The result of the QFT-G assay was defined as positive if IFN-γ level was≧0.35 IU/ml in TB-specific antigens-stimulated wells after subtracting the level of the nil well, according to the manufacturer’s recommendation. Positive QFT-G results were considered indicative of the patient having LTBI and an indicator of prophylactic therapy.

***Cells and mycobacteria culture***

The peripheral blood mononuclear cells (PBMCs) were immediately isolated from venous blood using Ficoll-Paque^TM^ PLUS (GE Healthcare Biosciences AB, Uppsala, Sweden) density gradient centrifugation. PBMCs or THP-1 cells were grown in RPMI medium supplemented with 10% FBS, 1x nonessential amino acids, 100 units/ml penicillin, and 100 units/ml streptomycin, in an incubator containing 5% CO_2_ at 37 °C. To induce differentiation, THP-1 cells (1.0 × 10^6^ cells) were grown in media and treated with 10 ng/ml phorbol myristate acetate (PMA; Sigma-Aldrich, USA) overnight. The THP-1 RFP-GFP-LC3 stable cell line (Difluo^TM^ hLC3) was purchased from InvivoGen (USA) and cultured on RPMI medium, according to the manufacturer’s protocol. The *M. bovis* BCG and *M. tuberculosis* H37Rv strain were cultured on Middlebrook 7H11 agar plates in an incubator containing 5% CO_2_ at 37 °C.

***MicroRNA next generation sequencing (NGS) analysis***

The small RNA library was prepared using a Total RNA-Seq kit v2.0 (ThermoFisher Scientific, USA). Template preparation was carried out with the Ion PGM Template OT2 200 kit (ThermoFisher Scientific, USA), according to the manufacturer’s protocol; the Ion PGM^TM^ Sequencing 200 kit (ThermoFisher Scientific, USA) and 318 chip were used with the Ion PGM sequencer, as described in the Ion PGM^TM^ Sequencing Kit User Guide. Data alignment to the hg19 human reference genome and base calling were done using the built-in Torrent Suite software v4.0 (ThermoFisher Scientific, USA). The differential expression analysis was carried out using Partek Genomic Suite 6.6 (Partek).

***Quantitative reverse transcription PCR (QRT-PCR)***

MicroRNA expression was measured and quantified using a TaqMan MicroRNA Assays kit (Applied Biosystems, ThermoFisher Scientific, USA), according to the manufacturer’s protocol. QRT-PCR reactions were performed on the StepOnePlus™ Real-Time PCR System (Applied Biosystems, ThermoFisher Scientific, USA), using a standard protocol. Each sample was run in triplicate. Small nuclear RNA (Rnu6, for cells) or synthetic cel-miR-39 (for plasma) was used as an internal control gene. The fold expression of the target gene relative to the averaged internal control gene in each sample was calculated using the comparative threshold cycle (Ct) method and evaluated by 2^dCt^, where dCt = mean of healthy controls (Ct _miRNAs gene_–Ct _Rnu6/cel-miR-39_) – patient (Ct _miRNAs gene_–Ct _Rnu6/cel-miR-39_).

***Transient transfection***

THP-1 cell-derived macrophages were transiently transfected with 30 nM miR-889 mimics (ThermoFisher Scientific, USA), TWEAK siRNA (Dharmacon, USA) or controls by using Lipofectamine RNAiMAX Transfection Reagent (ThermoFisher Scientific, USA) according to the manufacturer’s instructions, and incubated at 37°C for 24 h.

***Autophagosome maturation***

THP-1 cell-derived macrophages were grown on coverslips at a concentration of 1 × 10^5^ cells/well. TWEAK (100 ng/ml) or IFN-γ (200 U/ml) were added to cells for 24 h before infection. For autophagy inhibition, cells were incubated with 10 μM 3-MA for 6 h prior to adding TWEAK to block TWEAK-induced autophagy. Cells were infected with Texas red-labeled *M. bovis* BCG at a MOI of 10 for 1 h, washed three times with PBS to remove unbound mycobacteria, and incubated with LysoTracker Green (ThermoFisher Scientific, USA) or autophagic organelle specific fluorescent dye monodansylcadaverine (MDC) (Sigma-Aldrich, USA) for the final 1 h. Cells were fixed in 4% paraformaldehyde for 10 min at room temperature. Coverslips were mounted onto glass slides with mounting medium (ThermoFisher Scientific, USA), and images were recorded on an Olympus FV1000 laser scanning confocal microscope.

1. Aletaha D, Neogi T, Silman AJ, Funovits J, Felson DT, Bingham CO 3rd, Birnbaum NS, et al. (2010) 2010 rheumatoid arthritis classification criteria: an American College of Rheumatology/European League Against Rheumatism collaborative initiative. Ann. Rheum. Dis. *69*, 1580–8.
2. Prevoo ML, van 't Hof MA, Kuper HH, van Leeuwen MA, van de Putte LB, van Riel PL. (1995) Modified disease activity scores that include twenty-eight-joint counts. Development and validation in a prospective longitudinal study of patients with rheumatoid arthritis. [Arthritis Rheum.](http://www.ncbi.nlm.nih.gov/pubmed/?term=Frequency+of+infection+in+patients+with+rheumatoid+arthritis+compared+with+controls%3A+a+population-based+study.) *38*, 44–8.
3. Griffith DE, Aksamit T, Brown-Elliott BA, Catanzaro A, Daley C, Gordin F, Holland SM, Horsburgh R, Huitt G, Iademarco MF, et al. (2007) An official ATS/IDSA statement: diagnosis, treatment, and prevention of nontuberculous mycobacterial diseases. Am. J. Respir. Crit. Care Med. *175*, 367–416.
